# Comparative Proteomic Analysis of Cultured Suspension Cells of the Halophyte *Halogeton glomeratus* by iTRAQ Provides Insights into Response Mechanisms to Salt Stress

**DOI:** 10.3389/fpls.2016.00110

**Published:** 2016-02-09

**Authors:** Juncheng Wang, Lirong Yao, Baochun Li, Yaxiong Meng, Xiaole Ma, Yong Lai, Erjing Si, Panrong Ren, Ke Yang, Xunwu Shang, Huajun Wang

**Affiliations:** ^1^Gansu Provincial Key Lab of Aridland Crop Science/Gansu Key Lab of Crop Improvement and Germplasm EnhancementLanzhou, China; ^2^Department of Crop Genetics and Breeding, College of Agronomy, Gansu Agricultural UniversityLanzhou, China; ^3^Department of Botany, College of Life Science and Technology, Gansu Agricultural UniversityLanzhou, China; ^4^Department of Agriculture and Forestry, College of Agriculture and Animal Husbandry, Qinghai UniversityXining, China

**Keywords:** halophyte, *H. glomeratus*, iTRAQ, salt tolerance, cells, response mechanisms

## Abstract

Soil salinity severely threatens land use capability and crop yields worldwide. An analysis of the molecular mechanisms of salt tolerance in halophytes will contribute to the development of salt-tolerant crops. In this study, a combination of physiological characteristics and iTRAQ-based proteomic approaches was conducted to investigate the molecular mechanisms underlying the salt response of suspension cell cultures of halophytic *Halogeton glomeratus*. These cells showed halophytic growth responses comparable to those of the whole plant. In total, 97 up-regulated proteins and 192 down-regulated proteins were identified as common to both 200 and 400 mM NaCl concentration treatments. Such salinity responsive proteins were mainly involved in energy, carbohydrate metabolism, stress defense, protein metabolism, signal transduction, cell growth, and cytoskeleton metabolism. Effective regulatory protein expression related to energy, stress defense, and carbohydrate metabolism play important roles in the salt-tolerance of *H. glomeratus* suspension cell cultures. However, known proteins regulating Na^+^ efflux from the cytoplasm and its compartmentalization into the vacuole did not change significantly under salinity stress suggesting our existing knowledge concerning Na^+^ extrusion and compartmentalization in halophytes needs to be evaluated further. Such data are discussed in the context of our current understandings of the mechanisms involved in the salinity response of the halophyte, *H. glomeratus*.

## Introduction

Soil salinity severely limits robust plant growth and development, as well as the attainment of an adequate crop yield. It is estimated that approximately one-third of arable land throughout the world is affected by natural and secondary salinity to varying degrees (Munns, 2002, 2005; Munns and Tester, 2008; Li et al., 2012; Wang et al., 2013). Thus, improving the tolerance of crops to salinity in order to increase food production has become an urgent goal for plant breeders. Soil salinity mainly induces osmotic stress and ion toxicity, important processes that are considered most harmful to plants (Flowers and Colmer, 2008; Munns and Tester, 2008). For many plants, salt stress can cause a slowing of growth, wilting or even death. To survive under salinity stress, plants have evolved sophisticated response and adaptive mechanisms at biochemical, physiological, cellular, and molecular levels (Zhang et al., 2011). Many salt-tolerant-related candidate genes and gene products involved in salt uptake and transport (Munns et al., 2012; Guan et al., 2014), the elimination of reactive oxygen species (ROS; Suzuki et al., 2012; Peng et al., 2014), the accumulation of organic compounds (Ashraf and Foolad, 2007), the regulation of hormones (Jiang et al., 2013; Osakabe et al., 2014), and other processes have been conclusively identified. Unfortunately, however, salt-tolerant crops using some of these genes have not been developed due to the complexity of the mechanisms of salt tolerance (Witzel et al., 2010; Shabala and Munns, 2012). Understanding the molecular mechanisms of a salt response and defense in plants will help in the development of crops with salt tolerance (Deinlein et al., 2014; Munns and Gilliham, 2015). However, we have an extremely limited understanding of the molecular ion transport and regulatory mechanisms activated in plants under salt stress.

Halophytes are plants capable of surviving to reproduce in highly saline soils. Such a robust tolerance of salt is mainly due to the more effective control of the uptake and transport of Na^+^ and Cl^−^ compared with glycophytes (Flowers and Colmer, 2008). These special adaptive strategies make halophytes an excellent model to study molecular mechanisms of salt tolerance in plants. Recent advances in functional genomic technologies, including transcriptomics (Dang et al., 2013; Garg et al., 2013), proteomics (Wang et al., 2013, 2015b; Cheng et al., 2015), and metabolomics (Ruan and Teixeira da Silva, 2011; Obata and Fernie, 2012), have increased our knowledge of the complex regulatory networks associated with stress adaptation and tolerance of halophytes. Although changes in gene expression at the transcriptional level are not always reflected at the protein level (Lan et al., 2011), such biological processes are ultimately controlled by proteins. Therefore, clarification of changes in protein profiles using a proteomics approach will reveal a more realistic picture of the metabolic adjustments occurring during salinity stress. In the past two decades, a series of novel techniques have been developed and widely applied in the field of proteomics research. Isobaric tags for relative and absolute quantification (iTRAQ) is a powerful, gel-free quantification method that allows for the relative and absolute quantification of peptides. This technique is based on stable isotope labeling of peptides and measures peak intensities of reporter ions produced by precursor ion fragmentation in tandem mass spectrometry (Ross et al., 2004). In view of obvious advantages, such as high accuracy in identification and quantification of proteins, a sensitivity for low-abundance proteins, and the relatively high through-put multiplexing of up to eight samples (Bindschedler and Cramer, 2011), iTRAQ-based proteomics has been used to study several adaptive strategies of plants in response to abiotic challenges using leaves (Schneider et al., 2009; Liu et al., 2014), roots (Lan et al., 2011; Owiti et al., 2011; Wang et al., 2014), and suspension cell cultures (Rao et al., 2010; Böhmer and Schroeder, 2011). In an iTRAQ-based proteomics analysis on the mechanisms of a salinity response in halophytes, proteins involved in protein synthesis, photosynthesis, metabolism, and energy changed significantly under salt stress in *Thelungiella halophila* compared to *Arabidopsis thaliana* (Pang et al., 2010). Cheng et al. (2015) analyzed the dynamic protein expression patterns of leaves from *Tangut Nitraria* under salinity stress and showed that proteins related to redox homeostasis, photosynthesis, and energy metabolism made up the primary response networks to salinity (Cheng et al., 2015). However, this proteomics technique has not been used to explore the response mechanisms of the halophyte, *Halogeton glomeratus*, to salt stress.

The culture of plant cells in suspension offers a simplified model system for the study of cellular and molecular processes. Its predominant advantage is that a relatively homogenous, single cell population allows a rapid and uniform response to external stimuli, thereby avoiding the complications of multicellular types at the whole plant level (Mustafa et al., 2011). Based on the above advantages, suspension cell cultures have been widely used in investigating the physiological and molecular mechanisms involved in plant responses to salt stress. In their study of halophytes, Rosario Vera-Estrella et al. (1999) established a salt-tolerant, stable suspension culture of *Mesembryanthemum crystallinum* cells that showed a halophytic growth response comparable to that of the whole plant (Vera-Estrella et al., 1999). Their analysis of metabolic pathways suggested that NaCl stress induced programmed cell death in *T. halophila* suspension cell cultures that is similar to apoptosis in mammalian cells (Wang et al., 2010). Comparing ion content and distribution under NaCl stress in suspension cell cultures of the mangrove halophyte, *Sonneratia alba*, suggested that effective transport of Na^+^ and Cl^−^ into their vacuoles improved the salt tolerance of these cells (Hayatsu et al., 2014). Furthermore, during investigations of the salinity-induced proteomics of *Nitraria sphaerocarpa* suspension cells, it was found that proteins involved in signal transduction, cell rescue/defense, the cytoskeleton, the cell cycle, and in protein folding and assembly changed significantly after salt stress (Chen et al., 2012).

The halophyte, *H. glomeratus*, belongs to the Chenopodiaceae family, which has a strong tolerance to salinity; a physiological analysis of seedlings suggested that osmotic adjustment is one of the primary mechanisms of salt tolerance (Wang et al., 2015b). However, for osmotic adjustments in biological processes under salt stress, the primary transporters associated with Na^+^ accumulation in cells and its compartmentation in the vacuole remain are unclear (Wang et al., 2015a). The culture of salt-tolerant suspension cells is a useful tool for clarifying biological processes and primary transporters associated with Na^+^ accumulation and compartmentation under salt stress in halophytes (Vera-Estrella et al., 1999).

In the current study, we present a comprehensive proteomic analysis of suspension cell cultures of halophytic *H. glomeratus* treated with different NaCl concentrations using an iTRAQ-based approach. The objective of our work is to explore protein expression changes in response to NaCl and to highlight any potential response mechanisms of salt stress at a cellular level. This work will increase our understanding of halophyte cellular responses to salinity in *H. glomeratus* and will be of great interest in the rapidly developing field of salt-tolerant mechanisms. To our knowledge, this is the first report of the suspension cell culture of *H. glomeratus* in response to salinity stress using a comparative proteomics approach.

## Materials and methods

### Suspension cell cultures and salt stress treatments

Seedlings of *H. glomeratus* were used for generating calluses. Seeds of *H. glomeratus* were sterilized in 25% sodium hypochlorite and germinated on Murashige and Skoog (MS) solid medium under continuous light (300 μmol m^−2^ s^−1^) at 25°C and 70% relative humidity. For the 12 days culture, sterile apical meristems were cut off from seedlings and cultured on MS solid medium containing 2 mg/L 2,4-D (2,4-dichlorophenoxyacetic acid) at 25°C with continuous white light (100 μmol m^−2^ s^−1^). The medium was refreshed every 2 weeks. After 28 days, each 2.0 g of callus was transferred to 50 mL MS liquid medium containing 2 mg/L 2,4-D and grown in continuous white light (100 μmol m^−2^ s^−1^) with shaking at 120 rpm at 25°C. Cell suspensions were subcultured every 7 days for 5 weeks to obtain mainly synchronized cells. Salt stress treatments were performed, 7 days after changing the medium, by supplementing with 200 (moderate salinity stress) or 400 (severe salinity stress) mM NaCl. NaCl was not added to control medium. Cells were collected by filtration after the 5 days stress period (Whatman 113; Whatman International Ltd., Brentford, UK), frozen in liquid N_2_, and stored at −80°C for proteome analysis. Each treatment was made up of three biological replicates.

### Measurement of ion concentrations

Na^+^ and K^+^ contents was determined as described previously (Munns et al., 2010) using atomic absorption spectrometry. In brief, at the end of treatments, cells were rapidly rinsed three times with ultrapure water and freeze-dried at −50°C. Concentrations of Na^+^ or K^+^ were determined after digestion with 0.5% 0.5% nitric acid and the recovery of dry material as a fine powder.

### Cell viability assay

NaCl stress tolerance of cells was determined by assessing the relative cell viability of suspension cell cultures. Relative cell viability was measured using a 2,3,5-triphenyltetrazolium chloride (TTC) reduction method after a freeze-thaw cycle (Li et al., 2012). About 0.2 g of fresh cells per sample were used and three independent experiments were performed.

### Protein extraction and iTRAQ labeling

For protein extraction, cells were suspended in lysis buffer (7 M urea, 2 M thiourea, 4CHAPS, 40 mM Tris-HCl pH 8.5, 1 mM phenylmethylsulfonyl fluoride [PMSF], 2 mM ethylenediaminetetraacetic acid [EDTA]) and the mixture sonicated on ice. Samples were reduced with 10 mM dithiothreitol (DTT; final concentration) at 56°C for 1 h and then alkylated using 55 mM iodoacetamide (IAM; final concentration) for 45 min in darkness at room temperature. The reduced and alkylated protein mixtures were precipitated by adding a 4 × volume of chilled acetone at −20°C for 14 h. After centrifugation at 25,000 × g for 10 min at 4°C, the pellet was dissolved in 0.5 M tetraethylammonium borohydride (TEAB; Applied Biosystems, Milan, Italy) and sonicated on ice for 15 min. Proteins in the supernatant were collected after centrifuging at 25,000 × g at 4°C for 20 min, and protein concentrations determined by the Bradford method using bovine serum albumin as a standard (Bradford, 1976).

Protein (100 μg) was digested with 5 μg Trypsin Gold (Promega, Madison, WI, USA) at 37°C for 16 h. Peptides were reconstituted in 0.5 M TEAB and processed according to the manufacturer's protocol for 8-plex iTRAQ reagent (Applied Biosystems, Foster City, CA, USA). Each treatment was made up of two (Control) or three (Treated with 200 or 400 mM NaCl) biological replicates. Briefly, peptides were labeled with iTRAQ reagents 113 and 115 for control samples, 114, 116, and 118 for 200 mM NaCl-treated samples, and 117, 119, and 121 for 400 mM NaCl-treated samples, then pooled, and dried by vacuum centrifugation.

### Separation of peptides and LC-MS/MS analysis

Offline strong cation exchange (SCX) was performed as previously reported by Patterson et al. (2007) using a LC-20AB HPLC Pump system (Shimadzu, Kyoto, Japan) to fractionate complex peptides (Patterson et al., 2007). In total, SCX peptide fractions were pooled into 20 fractions, desalted using a Strata X C18 column (Phenomenex, Torrance, CA, USA) and vacuum-dried for liquid chromatography-tandem mass spectrometry (LC-MS/MS) analysis. Each dried fraction was resuspended in buffer (5% acetonitrile [ACN], 0.1% formic acid [FA]), centrifuged at 20,000 × g for 10 min; the final concentration of peptide was 0.5 μg/μL. A 5 μL peptide was loaded onto a LC-20AD nano high-pressure liquid chromatography analyzer (HPLC; Shimadzu, Kyoto, Japan) by an autosampler into a 2 cm C18 trap column (inner diameter 200 μm). Peptides were then eluted into a 10 cm analytical C18 column (inner diameter 75 μm) packed in-house.

Liquid chromatography-electrospray ionization-tandem mass spectrometry (LC-ESI-MS/MS) analysis of fractionated samples was performed using a TripleTOF 5600 System (AB SCIEX, Concord, ON, Canada), as described previously, at Beijing Genomics Institute (BGI, Shenzhen, China; Yang et al., 2013). An ion spray voltage of 2500 V was used for data acquisition. For information dependent acquisition (IDA), survey scans were acquired in 250 ms and as many as 30 product ion scans were collected if a threshold of 120 counts per second and with a charge state of 2+ to 5+ was exceeded. A sweeping collision energy of 35 eV was applied to all precursor ions for collision-induced dissociations.

### Database search and quantification

Protein identification and quantification were analyzed against a *H. glomeratus* protein database containing 30,124 non-redundant sequences (Source: NCBI [BioProject ID: PRJNA 254029]) using a Mascot search engine (Matrix Science, London, UK; version 2.3.02). For protein identification, the search parameters used were as follows: fragment mass tolerance of 0.1 Da, peptide mass tolerance of 0.05 Da, trypsin was chosen as the enzyme, with a maximum of one missed cleavage, Gln->pyro-Glu (N-term Q), Oxidation (M), Deamidated (NQ) as the potential variable modifications, and Carbamidomethyl (C), iTRAQ8plex (N-term), iTRAQ8plex (K) as fixed modifications. To reduce the probability of false peptide identification, only peptides at a 95% confidence interval (*P* < 0.05) with a false discovery rate (FDR) estimation ≤ 1.04% were counted as being successfully identified. Each positive protein identification contained at least one unique peptide. The MS-based proteomic data are available via ProteomeXchange with identifier PXD003358.

For protein quantitation, iTRAQ 8-plex was chosen for quantification during the search, and the minimum peptide was set to two. Proteins containing at least two unique spectra were selected for quantification analysis. The control sample was used as reference based on the weighted average of the intensity of reported ions in identified peptides. Quantitative protein ratios were weighted and normalized by the median ratio in Mascot. Ratios with *P* values < 0.05, and a >1.5-fold or < 0.67-fold cut-off were considered as up- or down-regulated proteins, respectively. Differentially expressed proteins were enriched to Gene Ontology (GO) Terms by the agriGO web service (http://bioinfo.cau.edu.cn/agriGO/index.php) on the basis of their biological functions. Protein classification was performed essentially on the basis of metabolic and functional features as described by Bevan et al. (1998).

## Results

### Effect of NaCl treatment on morphological and physiological changes

**Figure 1:**
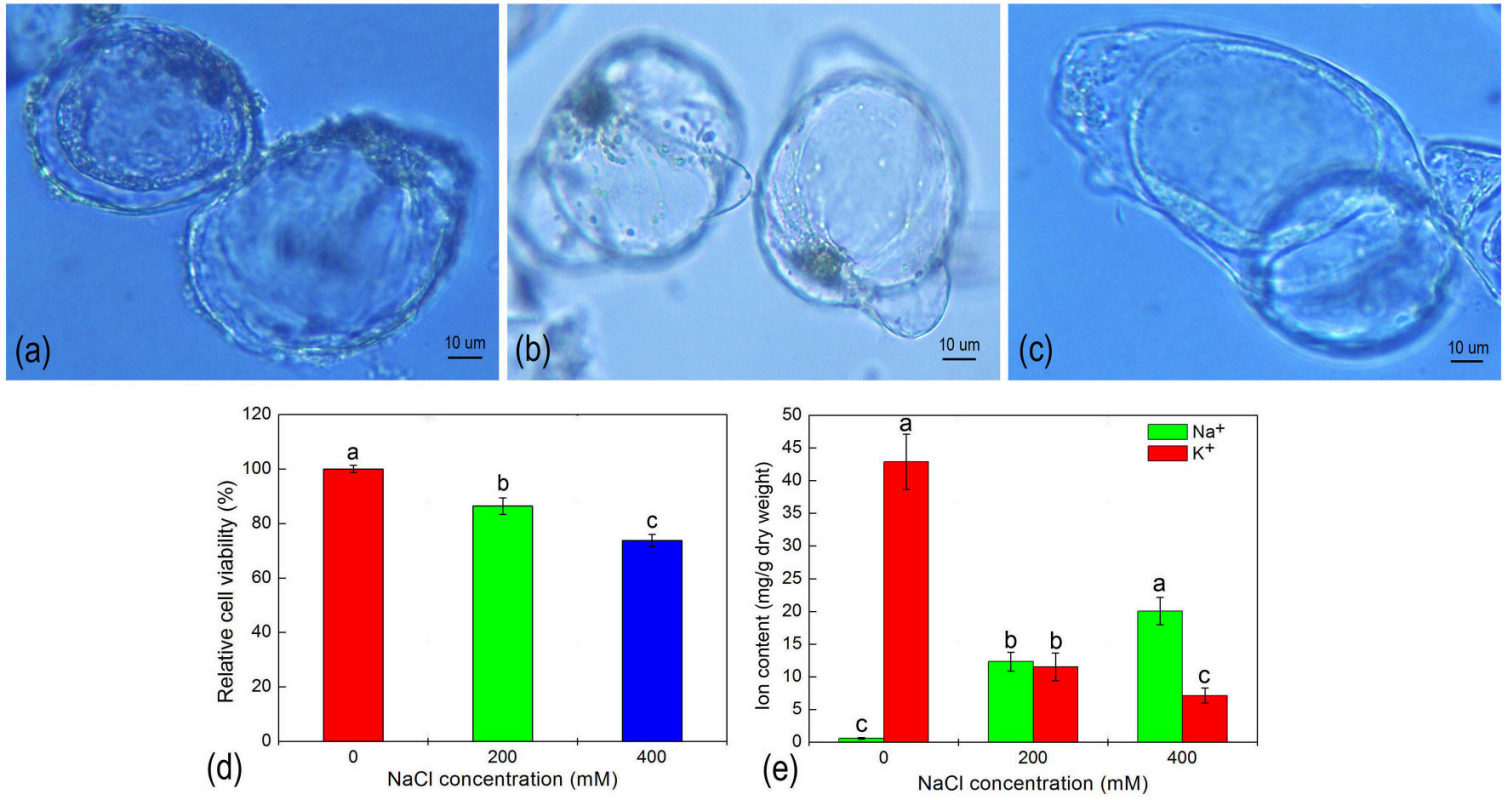
**The morphology (A–C), relative cell viability (D), and ion content (E) of ***H. glomeratus*** cells after treatment with different concentrations of NaCl for 5 d**. **(A)** 0 mM NaCl; **(B)** 200 mM NaCl; and **(C)** 400 mM NaCl. Values are expressed as the mean of three replicates ± standard error (SE). Means with the different letters indicate significant differences between different NaCl treatments according to Duncan's multiple range tests t (*p* < 0.05).

*H. glomeratus* is an extreme halophyte that compartmentalizes toxic ions into vacuoles as its primary salt-tolerance mechanism. In our studies, a large central vacuole was evident within suspension cells cultured with different salt treatments (Figures 1A–C). When *H. glomeratus* suspension cells were treated with 200 or 400 mM of NaCl for 5 d the size of the large central vacuole clearly confined the cytoplasm to a thin peripheral layer (Figures 1B,C). To evaluate the effect of ion toxicity on cell viability, we examined suspension cells cultured under different NaCl concentrations. As shown in Figure 1D, salt stress resulted in a significant inhibition of cell viability, showing a decrease to 86.39 and 73.74% of the control for 200 and 400 NaCl mM conditions, respectively (*P* < 0.05). When compared with control cells, the Na^+^ and K^+^ contents of cells significantly increased and decreased, respectively (Figure 1E). The Na^+^ concentration of cells treated with 200 or 400 mM NaCl were 20.18- or 32.81-fold higher than that of control cells. As a result, the K^+^/Na^+^ ratio in cells markedly decreased under salinity stress (Figure 1E). Thus, the analysis of these physiological features indicated that suspension cell cultures of *H. glomeratus* displayed a robust ability to tolerate NaCl stress similar to that observed at the whole plant level. The transport of Na^+^ across the plasma membrane and its segregation in the vacuole still play key roles in the regulation of ion homeostasis under salt stress in suspension cell cultures of *H. glomeratus*.

### Changes in suspension cell protein profiles under salt stress

**Figure 2:**
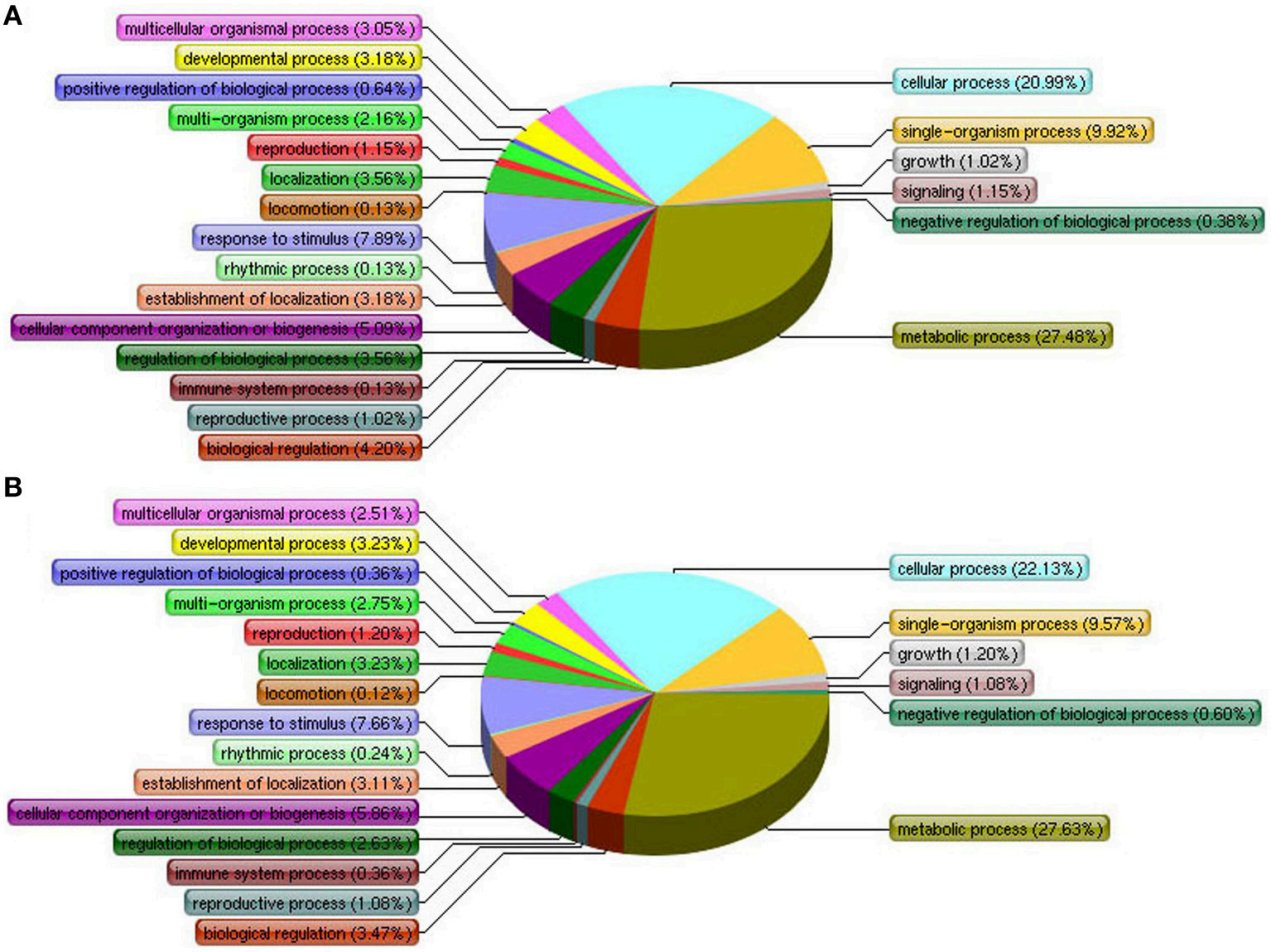
**Differentially expressed proteins of ***H. glomeratus*** cells are involved in primary biological processes according to Gene Ontology classifications. (A)** 200 mM NaCl and **(B)** 400 mM NaCl treatments.

To determine any proteomic changes in suspension cell cultures of *H. glomeratus* in response to NaCl stress at an adaptive stage, protein profiles for cells exposed to 0, 200, and 400 mM NaCl stress for 5 days were explored using an 8-plex iTRAQ method. At least two biological repeats were prepared for each condition. After analysis using mascot software by searching the total 444,052 spectra, a total of 23,893 peptides, and 5,649 proteins were identified. The detailed information of proteins and peptides is listed in Supplementary File 1. Proteins with at least two unique peptides (3,796, 67.2%) were quantified according to the criteria of a 95% confidence level (*P* < 0.05) and a 1.5-fold change upon up- or down-regulation. Compared to untreated control cells, 489 and 473 proteins changed significantly during 200 or 400 mM NaCl stress treatments, respectively (Supplementary Files 2, 3). The significantly enriched biological processes of these differentially expressed proteins were annotated by their GO annotation and enrichment analysis with AgriGO and are summarized in Figure 2 and Supplementary Files 4, 5. In 200 mM NaCl stressed cells, the prevalent categories included “metabolic process,” “cellular process,” “single-organism process,” “response to stimulus,” and “cellular component organization or biogenesis.” However, in 400 mM NaCl stressed cells, the number of proteins involved in “cellular process,” “metabolic process,” “cellular component organization or biogenesis,” and “multi-organism process” slightly increased, and the number of proteins involved in “regulation of biological process,” “biological regulation,” and “multicellular organismal process” decreased slightly when compared to protein categories associated with the 200 mM NaCl treatment (Figures 2A,B). This suggests that similar strategies might be activated in response to salt stress in suspension cells treated with Na^+^ concentrations ranging from 200 to 400 mM for 5 days.

### Potential proteins regulated by NaCl stress

**Figure 3:**
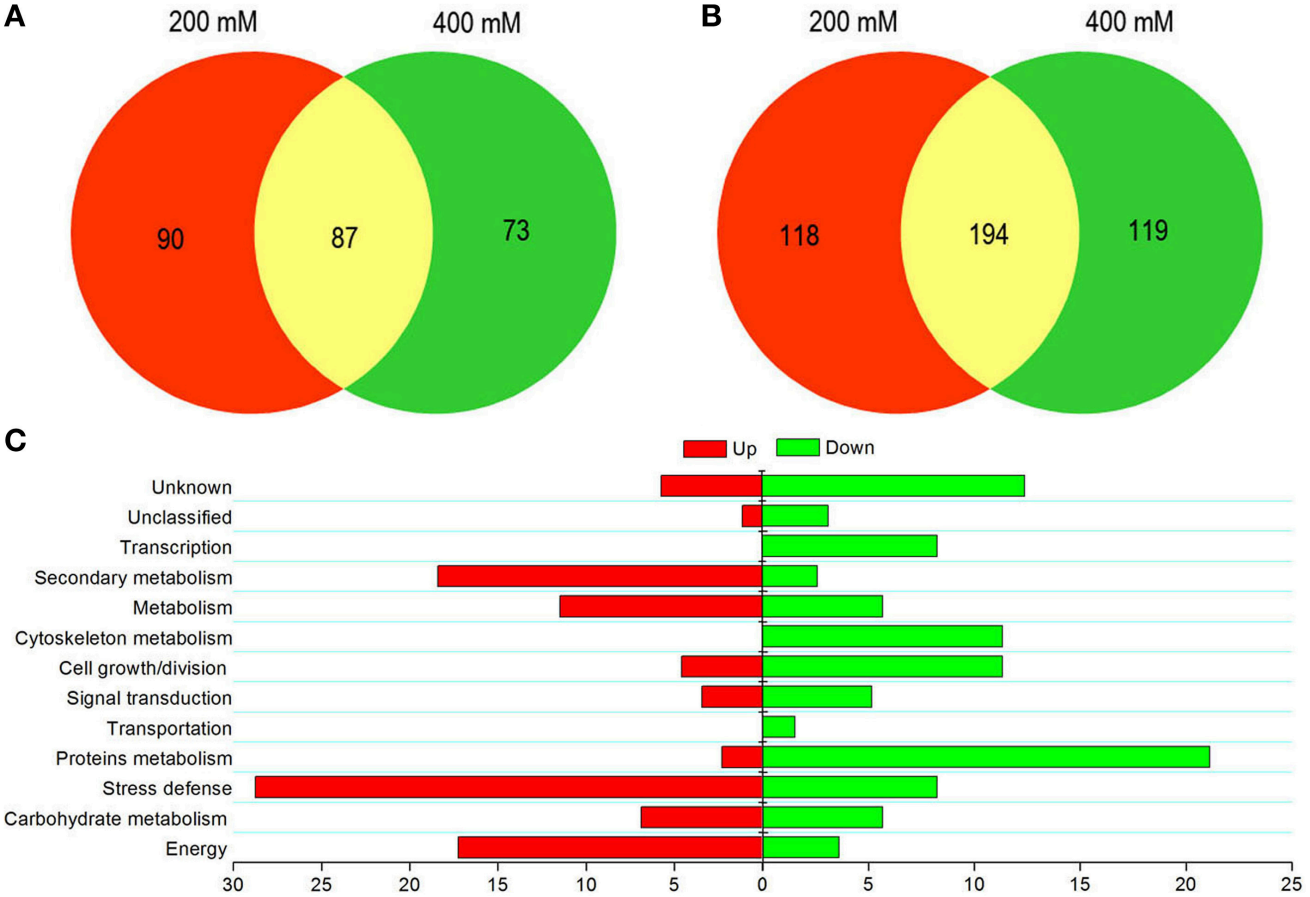
**Graphical representation and functional cataloging of differentially expressed proteins. Venn diagrams showing common, significantly up-regulated (A) and down-regulated proteins (B) in cells exposed to salt stress**. Functional classification of common up-regulated (left) and down-regulated (right) proteins based on Bevan et al. (1998) **(C)**. The histogram shows the distribution of the differentially expressed proteins into their functional classes represented by percentages.

The present study was concerned with identifying proteins involved in tolerance to salt stress and any associated tolerance mechanisms in suspension cell cultures. Of the differentially expressed proteins, 177 were up-regulated and 312 were down-regulated in the presence of 200 mM NaCl. Similarly, in 400 mM NaCl-treated cells, 160 proteins were up-regulated and 313 proteins were down-regulated. Notably, more down- than up-regulated proteins were identified when cells were exposed to different concentrations of NaCl stress. The numbers of differentially expressed proteins and how they overlapped under different NaCl treatments are illustrated by Venn diagram analysis as shown in Figure 3. Of 337 up-regulated proteins, 87 proteins were observed in cells under salinity stress at both NaCl concentrations (Figure 3A and Table S1). The functions of these proteins were classified into 10 categories, including energy (15, 17.24%), carbohydrate metabolism (6, 6.90%), stress defense (25, 28.74%), protein metabolism (2, 2.30%), signal transduction (3, 3.45%), cell growth/division (4, 4.60%), metabolism (10, 11.49%), secondary metabolism (16, 18.39%), unclassified (1, 1.15%), and unknown (5, 5.75%; Figure 3C). Of the 431 down-regulated proteins, 194 proteins were down-regulated when cells were exposed to both NaCl treatments (Figure 3B and Table S2). The functions of these down-regulated proteins were grouped into 13 categories involving energy (7, 3.16%), carbohydrate metabolism (11, 5.67%), stress defense (16, 8.28%), protein metabolism (41, 21.13%), transportation (3, 1.55%), signal transduction (10, 5.15%), cell growth/division (22, 11.34%), cytoskeleton metabolism (22, 11.34%), metabolism (11, 5.67%), secondary metabolism (5, 2.58%), transcription (16, 8.25%), unclassified (6, 3.09%), and unknown (24, 12.37%; Figure 3C).

In addition, a comparison between the differently expressed proteins and their categories was carried out (Figure 3C). The proportion of up-regulated proteins related to energy, carbohydrate metabolism, stress defense, metabolism, and secondary metabolism was higher than that of down-regulated proteins. However, the modulation of proteins involved in transportation and transcription was not observed at a 200 mM NaCl stress level (Figure 3C). This indicated that only high-salt stress condition significantly affected the biological processes of material transport and DNA transcription in suspension cells of *H. glomeratus*. In addition, many hypothetical proteins were detected among these differentially expressed proteins. Further studies are required on such proteins with unknown functions.

### Proteins related to toxic ion transport

**Figure 4:**
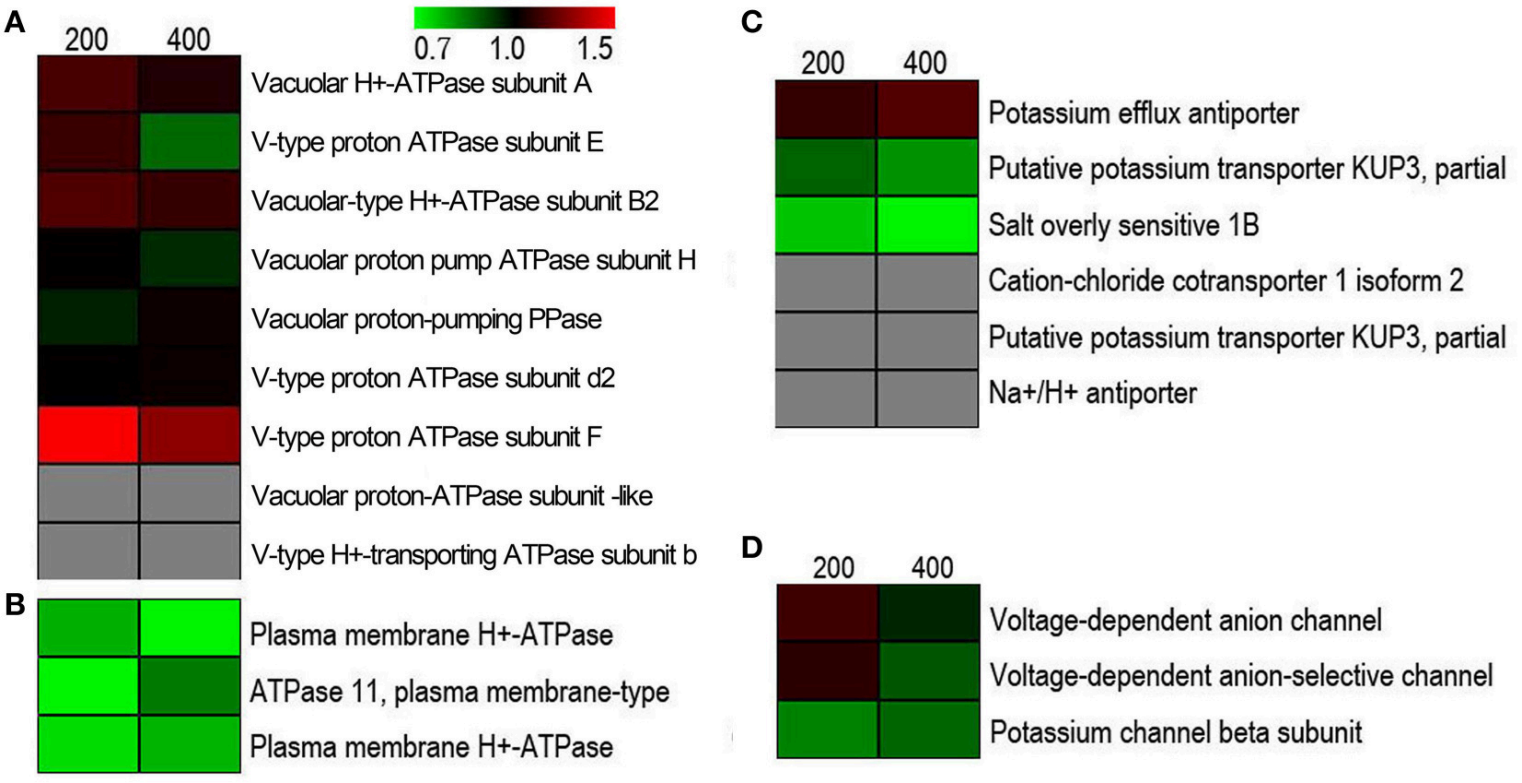
**Hierarchical clustering of salinity stress-responsive proteins involved in regulating Na^+^ and K^+^ homeostasis under salt stress. (A)** Tonoplast H^+^ pumps, **(B)** plasma membrane H^+^ pumps, **(C)** transporters, and **(D)** ion channels.

To investigate a possible ion-regulating model for proteins involved in transporting toxic ions (mainly Na^+^), we visualized the expression patterns of Na^+^ and K^+^ transport-related proteins in response to salt stress. As shown in Figure 4, a total of 21 proteins were identified related to Na^+^ and K^+^ transport, of which the category “tonoplast H^+^ pumps” represented the largest group (9, 42.85%), followed by “ion transporter” proteins (6, 28.57%), “plasma membrane H^+^ pumps” (3, 14.29%), and “channel” proteins (3, 14.29%). Contrary to our expectations, after using a cutoff value of a 1.5-fold-change threshold to stringently assess a protein as being responsive to salinity stress, none of these proteins showed markedly different expressions in all NaCl stress conditions compared to untreated cells. This indicated that ion transport in suspension cell cultures had achieved a state of balance in the extracellular space, cytoplasm, and vacuole after vacuole after being exposed to salinity for 5 d. Furthermore, ion transporter proteins, such as cation-chloride cotransporter 1 isoform 2, putative potassium transporter KUP3, and Na^+^/H^+^ antiporter, were only detected by one unique peptide, but its low expression abundance could not satisfy the quantitative study.

## Discussion

### Na^+^ accumulation and compartmentation in suspension cell cultures of *H. glomeratus*

We successfully established a suspension cell culture from seedlings of *H. glomeratus*. We then exposed suspension cell cultures to different NaCl stress conditions and analyzed any physiological changes at the cellular level. Suspension cells of *H. glomeratus* seedlings were capable of growing at high salinity levels, with their relative cell viability reaching up to 78% in 400 mM NaCl-containing medium compared to untreated, control cells. Moreover, an accumulation of Na^+^ was observed in salt-treated suspension cells, with the highest intracellular content of Na^+^ found when cells were grown in the presence of 400 mM NaCl for 5 d. These characteristics were similar to those reported previously for whole plants of *H. glomeratus* (Wang et al., 2015b) and related halophytes such as *M. crystallinum* (Vera-Estrella et al., 1999), and indicate that these cells are salt-tolerant and show growth characteristics in response to salinity comparable to those at the whole plant level. Although not all responses of cell suspensions to salinity were similar to those of whole plants, the large central vacuole that occupied most of the total cell volume, and high concentrations of Na^+^ accumulated within cells of salt-treated *H. glomeratus* are characteristic of whole plant responses to salinity stress (Figure 1). We hypothesize that a high concentration of Na^+^ was probably sequestered within the vacuole to avoid ion toxicity of the cytoplasm and to increase cellular osmolarity under salinity stress. The compartmentalization of Na^+^ into a vacuole is one of the most important strategies employed by suspension cell cultures of halophytes in response to salt stress (Vera-Estrella et al., 1999; Mimura et al., 2003).

### Protein identification and regulation under NaCl stress

The objective of our work was to identify response mechanisms of suspension cells to salt stress from the perspective of comparative proteomics. Liu et al. (2013) integrated information from proteomic and metabolomic studies of suspension cell cultures of rice under salinity stress. They demonstrated that salt-responsive networks of suspension cells were extremely complex and identical to those observed at the plant level (Liu et al., 2013). In our experiments, suspension cells of the halophyte, *H. glomeratus*, were treated with NaCl concentrations of 0, 200, and 400 mM for 5 days and then subjected to proteomics analysis using the gel-free iTRAQ system. Among 5,649 identified proteins, a total of 87 up- and 194 down-regulated proteins responded to both NaCl stress treatments (commonly responded). The functions of these salt responsive (mainly up-regulated) proteins and their main pathways are discussed in the following section.

### Proteins involved in energy and carbohydrate metabolism

A plant energy deficit is among the primary symptoms induced in response to salinity. The observed reduction in photosynthesis and/or respiration is closely associated with growth arrest and cell death (Baena-González and Sheen, 2008). We found that in suspension cells of *H. glomeratus*, 15 up-regulated proteins were noted after NaCl treatment, including ribulose bisphosphate carboxylase/oxygenases (Rubisco) activase (CL3673.Contig2 and CL3673.Contig4), ribulose bisphosphate carboxylase small chain 1 (Unigene1053), ribulose-phosphate 3-epimerase (Unigene20305) and phosphoribulokinase (Unigene25793), which are all Calvin cycle-specific enzymes. An increase in abundance of these proteins may contribute to an enhancement of Calvin cycle activity, leading to improvements of photosynthetic CO_2_ assimilation in response to salinity. Consistent with our previous proteome results at the whole-plant level, the relative abundance of Rubisco was a rapid increase in salt stress (Wang et al., 2015b). Two proteins involved in light reaction processes, namely, a 23 kDa precursor protein of the oxygen-evolving complex (Unigene6788), and a photosystem II reaction center PSB28 protein, have been shown to up-regulate light reaction-related proteins under NaCl stress in order to provide an adequate proton gradient and energy for other metabolic processes to operate normally (Pang et al., 2010). Furthermore, we also identified a serine hydroxymethyltransferase (Unigene11766) that belongs to a photorespiratory pathway.

The expression of electron transfer-related proteins, such as ferredoxin-1 (CL1018.Contig1, Unigene18531), a precursor of plastocyanin (Unigene13000), and glycerate dehydrogenase, (Unigene588) was up-regulated; these can modulate electron transfer effectively and improve ATPase synthesis and NADPH formation (Zhang et al., 2011). In addition, we identified an ATP-dependent zinc metalloprotease FTSH 2 (Unigene1270), a mitochondrial substrate carrier (Unigene14606), and an ATP synthase delta subunit precursor (Unigene523), which are classified as ATPase activity proteins. These results suggest that at high NaCl concentrations, suspension cells of *H. glomeratus* are able to deal with salt stress by sustaining appropriate level of the energy metabolism.

It is well established that carbohydrate metabolism largely changes in response to an energy shortage caused by salt stress in plants (Zhang et al., 2011). In our study, we, firstly, found a greater number of down- as opposed to up-regulated proteins involved in carbohydrate metabolism with salt treatment. Secondly, with regard to the down-regulated proteins, the abundance of most was lower for the 400 mM than the 200 mM NaCl treatment, indicating that normal pathways of carbohydrate metabolism were inhibited by a high concentration of NaCl. In suspension cells of *H. glomeratus*, four proteins belonging to the aldo-keto reductase (AKRS) superfamily, aldo-keto reductase yakc (CL1157.Contig1), aldo-keto reductase 2 (CL1157.Contig3, Unigene18105), and aldo/keto reductase (Unigene31104), were up-regulated under NaCl stress. AKRS, when induced by various stress treatments, confers tolerance to abiotic stresses (Éva et al., 2014; Kanayama et al., 2014) while overproduction of the OsAKR1 enzyme increases oxidative and heat stress tolerance through malondialdehyde and methylglyoxal detoxification in rice (Turóczy et al., 2011). The chloroplast glyceraldehyde-3-phosphate dehydrogenase (Unigene26663 and Unigene3804) was also increased in Na^+^-stressed suspension cells; this enzyme is not only involved in the glycolytic pathway, but also mediates plant metabolism and development under salt stress (Chang et al., 2015).

### Stress defense and signal transduction-related proteins

By regulating the expression of specific, stress-related genes and metabolites to counter ion imbalance, hyperosmotic stress, ROS production and oxidative damage (Huang et al., 2012), salt-induced defense responses, and signal transduction are crucial for salt tolerance. In our study, one of the most remarkable changes observed was the greater number of up-regulated proteins identified in stress defense after salt treatment. Of these, five proteins were determined to be pathogenesis-related proteins (PR proteins; CL3714.Contig2, CL4645.Contig1, Unigene1148, Unigene13991, and Unigene15500). Furthermore, the activation of six proteins: defense-related hydrolase chitinase 3 (CL3889.Contig1, Unigene26473), beta-1, 3-glucanase 31 (CL4645.Contig1), acidic endochitinase SP2 (CL39.Contig1), endochitinase PR4 (Unigene14744), and glycosyl hydrolase family 18 protein (CL3889.Contig2), was also detected after salt stress. PR proteins are usually induced by various abiotic and biotic stresses, which play a crucial role in plant defense (Liu and Ekramoddoullah, 2006). For instance, overexpression of PR protein genes enhanced tolerance to heavy metal and pathogen stresses (Sarowar et al., 2005) and to drought stress environments in tobacco (Jain et al., 2012).

Up-regulated glutathione transferase (Unigene22454, Unigene3942) and glutathione peroxidase (GSH-Px) are detoxifying enzymes, belonging to most families of the cell's antioxidant defense system. NaCl induces a quinone oxidoreductase-like protein (CL769.Contig1) that detoxifies quinones and their derivatives in leaves of salt-treated tomato plants (Zhou et al., 2009). Thaumatin-like protein (CL3555.Contig1) and osmotin-like protein (Unigene4336) are associated with osmotic adaption under salt stress (Ramos et al., 2015). A methionine sulfoxide reductase A4 (Unigene12027) repairs oxidized methionine and protects organisms from oxidative damage caused by ROS. In addition to these proteins, five stress response proteins, including putative prolyl aminopeptidase (Unigene15105), serine carboxypeptidase (Unigene25730), hypersensitive-induced response protein (Unigene2976) were identified as being up-regulated after salt stress.

Understanding salt-responsive signaling pathways is currently a hot topic in salt stress research. Here, three proteins, phosphatase 2C (CL2317.Contig1), proline-rich receptor-like protein kinase (CL3178.Contig2), and phospholipase D (CL5365.Contig1), were identified as having increased after NaCl stress. Previous reports have shown that a phosphatase 2C family protein phosphatase mediates ABA (abscisic acid)-regulated stomatal movement, water loss during leaf senescence, and abiotic stress tolerance in *Arabidopsis* (Zhang and Gan, 2012; Singh et al., 2015). The proline-rich receptor-like protein kinase belongs to the receptor-like protein kinase family, which may be part of a response to external challenges presented by an ever-changing environment (Morris and Walker, 2003). In short, the changes observed in this study suggest that proteins related to stress defense and signal transduction may be of particular importance for salt tolerance in cells.

### Proteins related to other metabolic systems

The balance between synthesis and degradation of proteins plays an important role in a plant's survival during abiotic adaptation (Hinkson and Elias, 2011). Generally, protein synthesis is inhibited by salt stress altering protein metabolic pathways (Zhang et al., 2011). From our iTRAQ data, we observed 44 proteins involved in protein translation, processing, and degradation were differently co-expressed after NaCl treatment. Of these differentially expressed proteins, only two were identified as up-regulated proteins, including aspartate aminotransferase (CL4696.Contig1), and elongation factor P (Unigene25772).

The cell's response to osmotic stress includes changing cell size and morphology to maintain turgor and this plays an important role in its survival under salt stress (Chen et al., 2012). We found that levels of the majority of cell growth and cytoskeleton-related proteins decreased in the presence of salinity (Tables S1,S2). The four up-regulated, growth-related proteins in response to NaCl stress were identified as auxin-induced atb2 (CL1157.Contig5), auxin-induced, protein PCNT115-like isoform 1 (CL6230.Contig2, Unigene17232), and indole-3-acetic acid-amido synthetase GH3.1-like (CL3188.Contig1). Of these, auxin-induced protein PCNT115-like isoform 1 was detected in a proteomics analysis in response to stimulation (Margaria et al., 2013) and development (Zhang et al., 2015). The indole-3-acetic acid-amido synthetase GH3.1 conjugates indole-3-acetic acid (IAA) to amino acids in an effort to maintain appropriate IAA concentrations in plants in order to regulate growth and development (Böttcher et al., 2010). Additionally, high intracellular Na^+^ levels evidently induced changes in the abundance of proteins involved in metabolism and secondary metabolism.

### Proteins related to ion homeostasis under salinity stress

**Figure 5:**
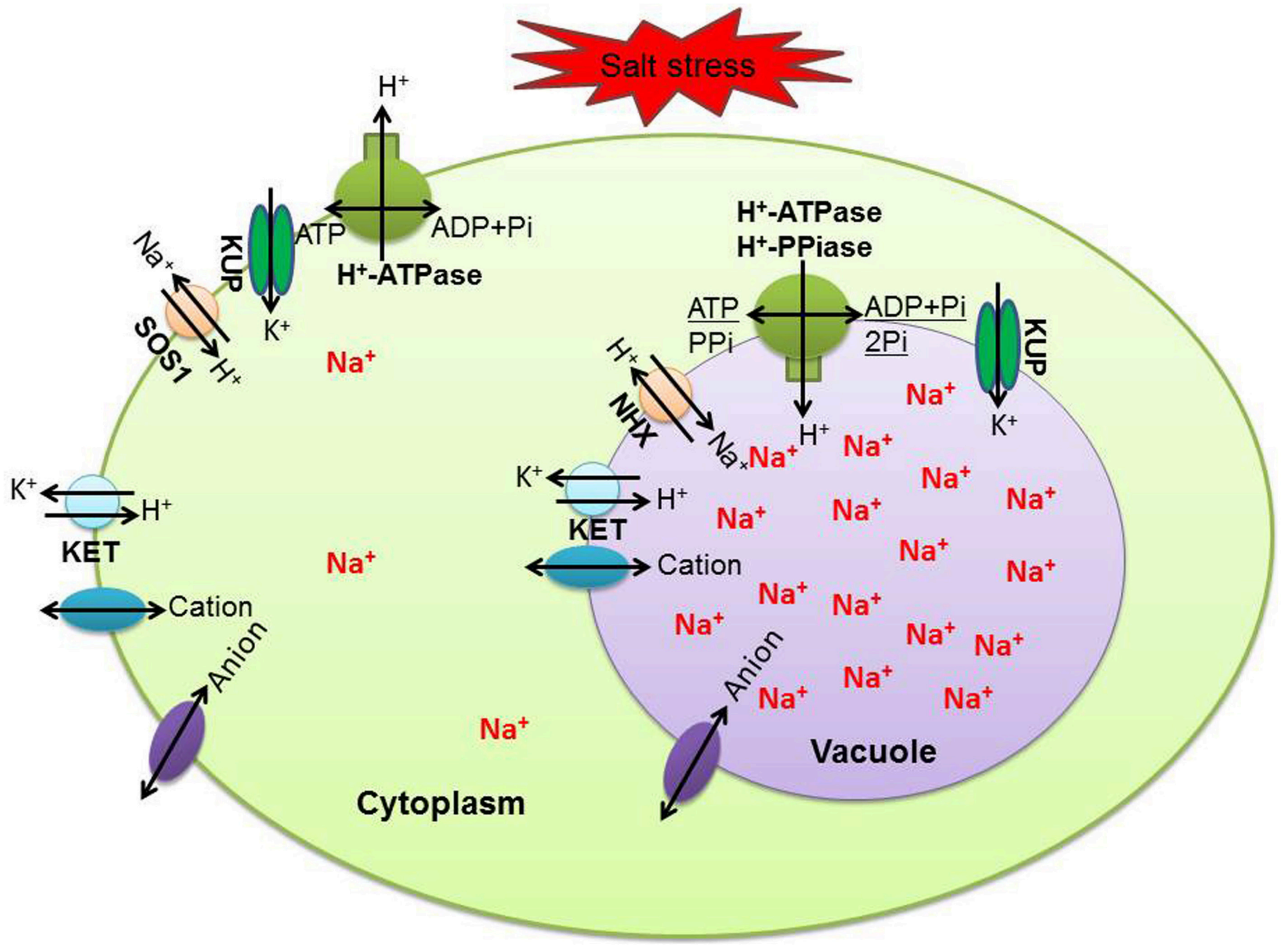
**Summary of key transporters and channels mediating Na^+^ and K^+^ homeostasis in ***H. glomeratus*** cells**. KUP, putative potassium transporter; SOS1, salt overly sensitive 1; KET, potassium efflux antiporter; NHX, Na^+^/H^+^ antiporter.

For ion homeostasis in the cell under salinity stress, ions that are extruded from the cytoplasm across the plasma membrane and compartmentalized in vacuoles cross the tonoplast using transporters or channels. This not only effectively alleviates the toxic effect of Na^+^ and Cl^−^, but also markedly increases cellular osmolarity to counter osmotic stress (Flowers and Colmer, 2008). Generally speaking, the plasma membrane Na^+^/H^+^ exchanger (*SOS 1*) and tonoplast Na^+^/H^+^ exchanger (*NHX*) are considered the main transporters mediating the efflux and compartmentalization of Na^+^ (Garcia de la Garma et al., 2015). Furthermore, increasing the activity of the H^+^-pump and V-ATPase in response to salinity correlates with an improvement in Na^+^/H^+^ antiport activity (Adolf et al., 2013). We have identified and summarized tonoplast H^+^ pumps (Figure 4A), plasma membrane H^+^ pumps (Figure 4B), transporters (Figure 4C), and channels (Figure 4D) related to transmembrane transport of ions in this study (Figure 4). Contrary to our expectations, none of these showed significantly changed abundances after salt stress. Of 21 proteins in the NaCl-stressed proteome, the tonoplast H^+^ pumps subfamily was the largest group, which probably provides energy required for vacuolar Na^+^ deposition; a relatively low abundance level of Na^+^/H^+^ antiporter was detected and this was partly consistent with our previous studies of the transcriptome (Wang et al., 2015a) and proteome (Wang et al., 2015b) of the salt stress response in *H. glomeratus*. Similarly, in *Arabidopsis*, tonoplast-localized *NHX* proteins mediate active potassium uptake into vacuoles but do not enhance the ability to compartmentalize Na^+^ in vacuoles (Barragán et al., 2012). Furthermore, for Na^+^ extrusion, the abundances of plasma protein H^+^-ATPase and *SOS1* were slightly decreased in salt stress. Obviously, the assumption of the plasma membrane Na^+^/H^+^ exchanger (*SOS 1*) and tonoplast Na^+^/H^+^ exchanger (*NHX*) mediating the efflux of Na^+^ from the cytoplasm and its compartmentalization into the vacuole is not in accordance with proteomic results (Figure 5 and Table S3). Meanwhile, recently in tobacco salt-acclimated cells, the subcellular analysis showed that Na^+^ compartmentalization in the cell vacuoles could be mediated through vesicle trafficking, so avoiding Na^+^ toxicity in the cytoplasm (Garcia de la Garma et al., 2015). Our quantitative proteomics data cannot fully explain the current view of Na^+^ extrusion and compartmentalization in *H. glomeratus*. Further work is needed to better understand the mechanisms of Na^+^ transport, including the Na^+^ efflux system of the plasma membrane and the Na^+^ uptake system of the tonoplast, using both functional assays and proteomics of subcellular compartments such as the tonoplast and plasma membrane proteomics analysis of *H. glomeratus* under salt stress.

## Conclusion

In conclusion, to our knowledge, this is the first report that identifies a large number of proteins associated with a salt response after treatment, with different concentrations of NaCl, of suspension cell cultures of the halophyte, *H. glomeratus*. Notably, suspension cell cultures of *H. glomeratus* showed a similar halophytic growth response to that of whole plants. We, here, have provided a comprehensive perspective on the salinity response of suspension cell cultures of *H. glomeratus* using a comparative proteomic approach. A total of 87 proteins were similarly up-regulated when exposed to two different NaCl concentrations; these proteins were mainly involved in energy, carbohydrate metabolism, stress defense, protein metabolism, signal transduction, cell growth, and cytoskeleton metabolism. In addition, known proteins that regulate the efflux of Na^+^ from the cytoplasm and its compartmentalization into the vacuole were not significantly changed under salinity stress. Whether suspension cells of *H. glomeratus* have specific transporters that are responsible for Na^+^ compartmentalization requires further study. Taken together, our results have contributed to crucial insights into the mechanisms involved in a salinity response by the halophytic *H. glomeratus*.

## Author contributions

JW and LY prepared suspension cell cultures of *H. glomeratus* samples for iTRAQ-based proteomic analysis. YL, BL, YM, and XM performed the general statistical analysis on the proteomics data. ES, KY, and PR participated in interpreting physiological characteristics results. JW, LY, and YL also involved in proteomics analysis and wrote the manuscript. HW and XS designed the experiment and provided guidance on the whole study. All authors have read and approved the final manuscript.

### Conflict of interest statement

The authors declare that the research was conducted in the absence of any commercial or financial relationships that could be construed as a potential conflict of interest.
