# Supplementary material for: Comparative Proteomic Analysis of Cultured Suspension Cells of the Halophyte Halogeton glomeratus by iTRAQ Provides Insights into Response Mechanisms to Salt Stress
Source: Front Plant Sci. 2016 Feb 9;7:110. doi: 10.3389/fpls.2016.00110 (PMC4746295; doi:10.3389/fpls.2016.00110)
Supplement: Supplementary file 1 [file Table1.doc]

Table S1 Common, significantly up-regulated proteins identified in *H. glomeratus* suspension culture cells grown in the presence of two different concentrations of NaCl.

| Accession  no^a^. | | Protein name | Species | Score | Coverage (%) | Peptides | Ratio^b^ | |
| --- | --- | --- | --- | --- | --- | --- | --- | --- |
|  |  |  |  |  |  |  | 200 | 400 |
| **Energy (15)** | | | | | | | | |
| CL1018.Contig1 | | PREDICTED: ferredoxin-1-like | *Vitis vinifera* | 170 | 9.7 | 1 | 1.922 | 2.118 |
| CL3673.Contig2 | | Ribulose bisphosphate Carboxylase/oxygenase activase, chloroplastic | *Spinacia oleracea* | 521 | 52.5 | 1 | 2.501 | 2.113 |
| CL3673.Contig4 | | Ribulose bisphosphate carboxylase activase | *Lemna minor* | 2676 | 46.6 | 11 | 1.505 | 1.64 |
| CL6196.Contig2 | | PREDICTED: photosystem II reaction center Psb28 protein, chloroplastic-like | *Fragaria vesca subsp. vesca* | 151 | 8.6 | 1 | 1.857 | 1.615 |
| Unigene1053 | | Ribulose bisphosphate carboxylase small chain 1, chloroplastic; Short=RuBisCO small subunit 1; | *Mesembryanthemum crystallinum* | 557 | 31.7 | 5 | 1.522 | 2.063 |
| Unigene11766 | | Serine hydroxymethyltransferase | *Zea mays* | 2176 | 39.5 | 13 | 1.739 | 1.757 |
| Unigene1270 | | ATP-dependent zinc metalloprotease FTSH 2, chloroplastic | *Oryza sativa subsp. japonica* | 259 | 23.1 | 3 | 1.686 | 1.683 |
| Unigene13000 | | Plastocyanin, chloroplastic; Flags: Precursor | *Spinacia oleracea* | 264 | 23.6 | 2 | 2.087 | 1.575 |
| Unigene14606 | | Mitochondrial substrate carrier | *Paeonia suffruticosa* | 896 | 33.1 | 10 | 2.008 | 2.015 |
| Unigene18531 | | PREDICTED: ferredoxin-1 | *Vitis vinifera* | 77 | 21.8 | 2 | 1.569 | 1.594 |
| Unigene20305 | | Ribulose-phosphate 3-epimerase | *Spartina alterniflora* | 1164 | 21.1 | 4 | 1.572 | 1.718 |
| Unigene25793 | | Phosphoribulokinase | *Beta vulgaris* | 597 | 19.5 | 8 | 1.649 | 1.788 |
| Unigene523 | | ATP synthase delta subunit precursor | *Spinacia oleracea* | 409 | 26.9 | 5 | 1.644 | 2.324 |
| Unigene588 | | PREDICTED: glycerate dehydrogenase HPR, peroxisomal | *Solanum lycopersicum* | 489 | 27.2 | 7 | 1.578 | 1.852 |
| Unigene6788 | | 23 kDa precursor protein of the oxygen-evolving complex | *Salicornia europaea* | 991 | 29.1 | 6 | 1.78 | 2.077 |
| **Carbohydrate metabolism (6)** | | | | | | | | |
| CL1157.Contig1 | | Aldo-keto reductase yakc | *Medicago truncatula* | 5141 | 55.3 | 9 | 1.866 | 1.772 |
| CL1157.Contig3 | | PREDICTED: probable aldo-keto reductase 2-like | *Fragaria vesca subsp. vesca* | 3160 | 25 | 3 | 1.729 | 1.546 |
| Unigene18105 | | Probable aldo-keto reductase 2 | *Oryza sativa Indica Group* | 2075 | 55.1 | 7 | 2.4 | 1.987 |
| Unigene26663 | | Glyceraldehyde-3-phosphate dehydrogenase B, chloroplastic | *Vicia sativa* | 1520 | 50.5 | 7 | 1.514 | 1.593 |
| Unigene31104 | | Aldo/keto reductase | *Medicago truncatula* | 775 | 33.7 | 1 | 4.415 | 2.798 |
| Unigene3804 | | Glyceraldehyde-3-phosphate dehydrogenase A, chloroplastic | *Helianthus annuus* | 4314 | 39.2 | 14 | 1.847 | 2.019 |
| **Stress defense (25)** | | | | | | | | |
| CL3555.Contig1 | | Thaumatin-like protein | *Mirabilis jalapa* | 543 | 28.1 | 4 | 2.082 | 1.956 |
| CL3714.Contig2 | | Pathogenesis-related protein R | *Nicotiana tabacum* | 588 | 30.1 | 5 | 2.739 | 2.307 |
| CL3889.Contig1 | | Chitinase 3 | *Populus x canadensis* | 1209 | 27.5 | 7 | 2.998 | 2.211 |
| CL3889.Contig2 | | Glycosyl hydrolase family 18 protein | *Arabidopsis lyrata subsp. lyrata* | 741 | 39.1 | 4 | 1.878 | 1.701 |
| CL39.Contig1 | | Acidic endochitinase SP2 | *Beta vulgaris* | 1898 | 29.1 | 5 | 3.385 | 2.371 |
| CL4645.Contig1 | | Beta-1,3-glucanase 31 | *Solanum tuberosum* | 171 | 10.8 | 2 | 2.706 | 3.959 |
| CL769.Contig1 | | PREDICTED: quinone oxidoreductase-like protein At1g23740, chloroplastic-like | *Solanum lycopersicum* | 169 | 19 | 5 | 1.686 | 1.61 |
| Unigene1148 | | Pathogenesis-related protein | *Spinacia oleracea* | 769 | 63.2 | 4 | 1.881 | 2.233 |
| Unigene12027 | | Methionine sulfoxide reductase A4 | *Solanum lycopersicum* | 184 | 35.8 | 3 | 1.524 | 1.557 |
| Unigene13908 | | Pleiotropic drug resistance protein 1 | *Nicotiana tabacum* | 711 | 27.5 | 3 | 3.095 | 2.218 |
| Unigene13991 | | Pathogenesis-related protein | *Spinacia oleracea* | 194 | 29.3 | 2 | 1.895 | 1.743 |
| Unigene14744 | | Endochitinase PR4 | *Phaseolus vulgaris* | 403 | 9 | 2 | 2.853 | 1.694 |
| Unigene15105 | | Putative prolyl aminopeptidase | *Oryza sativa Japonica Group* | 311 | 19.4 | 4 | 3.536 | 2.217 |
| Unigene15500 | | Pathogenesis-related protein 1 | *Vitis hybrid cultivar* | 429 | 28.7 | 3 | 5.257 | 3.314 |
| Unigene17451 | | 4-coumarate--CoA ligase-like 5 | *Arabidopsis thaliana* | 173 | 14.5 | 7 | 1.512 | 1.54 |
| Unigene17647 | | Salinity-induced protein | *Alternanthera philoxeroides* | 1108 | 39.9 | 8 | 2.002 | 1.539 |
| Unigene20325 | | Glutathione peroxidase | *Malus x domestica* | 266 | 22.9 | 4 | 1.823 | 1.645 |
| Unigene20547 | | PREDICTED: proline iminopeptidase-like | *Vitis vinifera* | 972 | 35.2 | 8 | 3.078 | 1.826 |
| Unigene22454 | | Glutathione transferase GST 23 | *Zea mays* | 46 | 8.4 | 2 | 2.549 | 1.625 |
| Unigene25730 | | PREDICTED: serine carboxypeptidase-like 45-like | *Vitis vinifera* | 1485 | 39 | 10 | 3.187 | 2.541 |
| Unigene26473 | | Chitinase 3 | *Populus x canadensis* | 254 | 38.3 | 3 | 2.432 | 1.641 |
| Unigene2976 | | Hypersensitive-induced response protein 1 | *Carica papaya* | 399 | 23.7 | 3 | 1.658 | 2.265 |
| Unigene3908 | | Small heat shock protein, partial | *Galium verum var. asiaticum* | 672 | 30.9 | 6 | 1.552 | 1.592 |
| Unigene3942 | | Glutathione transferase | *Suaeda maritima* | 557 | 34.8 | 7 | 2.958 | 1.954 |
| Unigene4336 | | Osmotin-like protein | *Atriplex nummularia* | 270 | 28.6 | 4 | 2.618 | 2.113 |
| **Proteins metabolism (2)** | | | | | | | | |
| CL4696.Contig1 | | Aspartate aminotransferase | *Prunus persica* | 9320 | 60.9 | 18 | 1.648 | 1.661 |
| Unigene25772 | | Elongation factor P | *Synechococcus elongatus* | 179 | 15.4 | 3 | 1.861 | 1.828 |
| **Signal transduction (3)** | | | | | | | | |
| CL2317.Contig1 | | Probable protein phosphatase 2C 39 | *Arabidopsis thaliana* | 381 | 32.6 | 6 | 2.759 | 1.785 |
| CL3178.Contig2 | | Putative proline-rich receptor-like protein kinase | *Arabidopsis thaliana* | 120 | 5.9 | 4 | 1.953 | 2.027 |
| CL5365.Contig1 | | Phospholipase D | *Pyrus pyrifolia* | 4716 | 44.7 | 25 | 1.626 | 1.553 |
| **Cell growth/division (4)** | | | | | | | | |
| CL1157.Contig5 | | Auxin-induced atb2 | *Arabidopsis thaliana* | 2520 | 57.6 | 1 | 2.192 | 1.614 |
| CL3188.Contig1 | | PREDICTED: probable indole-3-acetic acid-amido synthetase GH3.1-like | *Glycine max* | 1095 | 28.7 | 13 | 2.734 | 3.001 |
| CL6230.Contig2 | | PREDICTED: auxin-induced protein PCNT115-like isoform 1 | *Glycine max* | 1910 | 47.1 | 2 | 3.312 | 2.123 |
| Unigene17232 | | PREDICTED: auxin-induced protein PCNT115 isoform 1 | *Vitis vinifera* | 4289 | 51 | 13 | 1.737 | 1.782 |
| **Metabolism (10)** | | | | | | | | |
| CL3810.Contig2 | | Acyl-coenzyme A oxidase 3,  peroxisomal | *Arabidopsis thaliana* | 1561 | 36.6 | 2 | 1.661 | 1.74 |
| CL4132.Contig2 | | 2-oxoglutarate/Fe(II)-dependent dioxygenase | *Papaver somniferum* | 323 | 21.8 | 6 | 5.041 | 2.92 |
| CL2820.Contig2 | | PREDICTED: probable Plastid-lipid-associated protein 13, chloroplastic-like | *Fragaria vesca subsp. vesca* | 582 | 43.6 | 9 | 1.512 | 1.581 |
| CL5483.Contig2 | | Probable plastid-lipid-associated protein 5, chloroplastic | *Arabidopsis thaliana* | 438 | 47.6 | 7 | 1.616 | 1.517 |
| Unigene15421 | | Phenylalanine ammonia-lyase  class 2 | *Phaseolus vulgaris* | 350 | 44.3 | 4 | 2.236 | 1.861 |
| Unigene15931 | | Epoxide hydrolase, putative | *Ricinus communis* | 349 | 32.9 | 5 | 2.12 | 1.855 |
| Unigene19827 | | AMP-dependent CoA ligase, putative | *Ricinus communis* | 819 | 20.7 | 10 | 2.683 | 2.431 |
| Unigene31542 | | PREDICTED: probable linoleate 9S-lipoxygenase 5-like | *Glycine max* | 463 | 60.9 | 4 | 2.2 | 2.888 |
| Unigene489 | | PI-PLC X domain-containing protein At5g67130 | *Arabidopsis thaliana* | 718 | 16.9 | 7 | 1.521 | 1.578 |
| Unigene715 | | Acyl-CoA oxidase, putative | *Ricinus communis* | 1666 | 38.6 | 19 | 1.667 | 1.507 |
| **Secondary metabolism (16)** | | | | | | | | |
| CL102.Contig2 | Secologanin synthase | | *Catharanthus roseus* | 636 | 24.1 | 9 | 1.914 | 1.553 |
| CL160.Contig6 | Cytochrome P450 | | *Populus trichocarpa* | 520 | 29.5 | 7 | 1.955 | 2.195 |
| CL2504.Contig1 | O-methyltransferase | | *Vitis vinifera* | 285 | 18.1 | 5 | 2.024 | 3.492 |
| CL2504.Contig3 | O-methyltransferase | | *Vitis vinifera* | 1098 | 34.5 | 5 | 2.616 | 1.506 |
| CL2796.Contig2 | 4,5-DOPA dioxygenase extradiol | | *Amaranthus hypochondriacus* | 307 | 17.4 | 4 | 2.417 | 1.7 |
| CL4066.Contig1 | PREDICTED: cytochrome P450 83B1-like | | *Solanum lycopersicum* | 607 | 25.8 | 10 | 1.937 | 1.672 |
| CL4871.Contig2 | Cinnamyl alcohol dehydrogenase | | *Punica granatum* | 930 | 42.2 | 3 | 1.973 | 1.586 |
| CL619.Contig3 | Cytochrome P450 71A1 | | *Vitis vinifera* | 597 | 21.1 | 8 | 2.677 | 1.87 |
| Unigene12435 | Chalcone isomerase | | *Litchi chinensis* | 1395 | 51.9 | 9 | 2.928 | 2.097 |
| Unigene17518 | 2-C-methyl-D-erythritol 4-phosphate cytidylyltransferase, chloroplastic | | *Oryza sativa subsp. japonica* | 220 | 16.1 | 4 | 1.636 | 1.738 |
| Unigene20518 | Reticuline oxidase-like protein | | *Arabidopsis thaliana* | 602 | 43.5 | 7 | 1.545 | 2.084 |
| Unigene23338 | Caffeic acid 3-O-methyltransferase | | *Prunus dulcis* | 2559 | 50.4 | 13 | 2.279 | 2.004 |
| Unigene27016 | Cytochrome P450 94A2 | | *Vicia sativa* | 197 | 43.2 | 5 | 1.749 | 1.765 |
| Unigene34347 | PREDICTED: cytochrome P450 94A1-like | | *Vitis vinifera* | 888 | 40.4 | 5 | 2.128 | 2.245 |
| Unigene6843 | Indole-2-monooxygenase | | *Zea mays* | 440 | 18.1 | 2 | 3.587 | 2.534 |
| Unigene9481 | Reticuline oxidase precursor, putative | | *Ricinus communis* | 163 | 18.9 | 3 | 1.998 | 3.265 |
| **Unclassified (1)** | | | | | | | | |
| Unigene9763 | | PREDICTED: minor allergen Alt a 7-like | *Vitis vinifera* | 1762 | 42.3 | 8 | 2.07 | 1.73 |
| **Unknown (5)** | | | | | | | | |
| Unigene14620 | | Hypothetical protein PRUPE_ppa009927mg | *Prunus persica* | 148 | 6.2 | 1 | 1.823 | 1.792 |
| Unigene20333 | | Conserved hypothetical protein | *Ricinus communis* | 285 | 30 | 4 | 1.746 | 1.861 |
| Unigene3660 | | Predicted protein | *Populus trichocarpa* | 102 | 9.4 | 1 | 2.219 | 1.722 |
| Unigene537 | | Unknown | *Lotus japonicus* | 476 | 61.8 | 6 | 1.737 | 2.123 |
| Unigene729 | | Predicted protein | *Populus trichocarpa* | 147 | 22 | 5 | 1.815 | 1.592 |

^a^Accession no. according to EST database of *H.glomeratus*.

^b^The values were calculated as the ratio between intensities of identified protein in treatments (200, and 400 mM NaCl) vs. control (0 mM NaCl)
