# Supplementary material for: Comparative Proteomic Analysis of Cultured Suspension Cells of the Halophyte Halogeton glomeratus by iTRAQ Provides Insights into Response Mechanisms to Salt Stress
Source: Front Plant Sci. 2016 Feb 9;7:110. doi: 10.3389/fpls.2016.00110 (PMC4746295; doi:10.3389/fpls.2016.00110)
Supplement: Supplementary file 2 [file Table2.doc]

Table S2. Common, significantly down-regulated proteins identified in *H. glomeratus* suspension cell cultures grown in different NaCl concentrations.

| Accession  no^a^. | Protein name | Species | Score | Coverage (%) | Peptides | Ratio^b^ | |
| --- | --- | --- | --- | --- | --- | --- | --- |
|  |  |  |  |  |  | 200 mM | 400 mM |
| **Energy (7)** | | | | | | | |
| CL2156.Contig2 | PREDICTED: dihydrolipoyllysine-residue acetyltransferase component of pyruvate dehydrogenase complex, mitochondrial-like | *Vitis vinifera* | 436 | 19.1 | 5 | 0.514 | 0.659 |
| CL3195.Contig1 | PREDICTED: kidney mitochondrial carrier protein 1-like | *Vitis vinifera* | 559 | 25.5 | 6 | 0.542 | 0.481 |
| CL3478.Contig2 | PREDICTED: pyruvate dehydrogenase E1 component subunit beta-like isoform 2 | *Cucumis sativus* | 307 | 9.8 | 3 | 0.488 | 0.539 |
| CL4248.Contig2 | PREDICTED: pyruvate dehydrogenase E1 component subunit alpha | *Vitis vinifera* | 488 | 35.5 | 10 | 0.49 | 0.515 |
| Unigene3232 | ATP binding protein, putative | *Ricinus communis* | 140 | 5.1 | 5 | 0.484 | 0.519 |
| Unigene5901 | ATP binding protein, putative | *Ricinus communis* | 158 | 8.3 | 4 | 0.536 | 0.555 |
| Unigene891 | PREDICTED: dihydrolipoyllysine-residue acetyltransferase component of pyruvate dehydrogenase complex, mitochondrial-like isoform 1 | *Vitis vinifera* | 275 | 25 | 6 | 0.59 | 0.598 |
| **Carbohydrate metabolism (11)** | | | | | | | |
| CL4583.Contig1 | Alpha-glucosidase, putative | *Medicago sativa* | 1586 | 28.2 | 18 | 0.564 | 0.465 |
| CL5397.Contig3 | Beta-glucosidase, putative | *Ricinus communis* | 176 | 9.3 | 4 | 0.652 | 0.594 |
| Unigene1077 | PREDICTED: pyrophosphate--fructose 6-phosphate 1-phosphotransferase subunit alpha-like | *Ricinus communis* | 1102 | 21.1 | 6 | 0.658 | 0.586 |
| Unigene14814 | Pyruvate kinase isozyme G, chloroplastic-like | *Cucumis sativus* | 127 | 11.2 | 3 | 0.445 | 0.533 |
| Unigene15126 | dTDP glucose-4,6-dehydratase | *Theobroma cacao* | 778 | 37.6 | 8 | 0.555 | 0.609 |
| Unigene17688 | UDP-glucose 6-dehydrogenase | *Glycine max* | 865 | 31.7 | 9 | 0.62 | 0.59 |
| Unigene208 | Beta-galactosidase 3-like | *Vitis vinifera* | 194 | 7.9 | 5 | 0.634 | 0.593 |
| Unigene25497 | Endo-1,4-beta-glucanase isoform 05 | *Fragaria x ananassa* | 603 | 22.6 | 8 | 0.533 | 0.437 |
| Unigene493 | PREDICTED: lysosomal beta glucosidase-like | *Solanum lycopersicum* | 633 | 18 | 6 | 0.311 | 0.275 |
| Unigene6727 | UDP-glucose 6-dehydrogenase | *Glycine max* | 1571 | 49 | 10 | 0.551 | 0.544 |
| Unigene9185 | PREDICTED: endoglucanase 24 | *Vitis vinifera* | 130 | 8.7 | 4 | 0.467 | 0.399 |
| **Stress defense (16)** | | | | | | | |
| CL1379.Contig1 | C2 domain-containing protein | *Cicer arietinum* | 838 | 14.2 | 21 | 0.574 | 0.626 |
| CL4548.Contig2 | PREDICTED: peroxidase 52-like isoform 2 | *Glycine max* | 321 | 27.2 | 6 | 0.468 | 0.54 |
| CL4563.Contig2 | Thiamine thiazole synthase, chloroplastic | *Populus euphratica* | 356 | 9.7 | 2 | 0.342 | 0.405 |
| Unigene12045 | Multicopper oxidase, putative | *Ricinus communis* | 485 | 18.1 | 5 | 0.446 | 0.341 |
| Unigene12273 | Protein auxin response 4 | *Arabidopsis thaliana* | 284 | 12.9 | 5 | 0.62 | 0.661 |
| Unigene12685 | Disease-resistance protein SGT1 | *Saccharum officinarum* | 157 | 14.6 | 4 | 0.616 | 0.611 |
| Unigene1464 | PREDICTED: bifunctional polymyxin resistance protein ArnA-like | *Solanum lycopersicum* | 1110 | 47.9 | 11 | 0.504 | 0.558 |
| Unigene14929 | PREDICTED: thaumatin-like protein-like | *Vitis vinifera* | 88 | 10.8 | 2 | 0.426 | 0.334 |
| Unigene15079 | GDSL-lipase | *Oxybasis rubra* | 276 | 21.4 | 6 | 0.402 | 0.383 |
| Unigene1540 | PREDICTED: heat shock factor-binding protein 1-like | *Fragaria vesca subsp. vesca* | 316 | 53.4 | 3 | 0.544 | 0.509 |
| Unigene22238 | Epoxide hydrolase | *Medicago truncatula* | 64 | 14.4 | 2 | 0.417 | 0.454 |
| Unigene23689 | Major latex like protein homolog | *Beta vulgaris* | 224 | 23.5 | 3 | 0.494 | 0.432 |
| Unigene23974 | STS14 protein | *Solanum tuberosum* | 268 | 22.4 | 3 | 0.659 | 0.618 |
| Unigene6156 | PREDICTED: L-ascorbate oxidase homolog | *Solanum lycopersicum* | 318 | 18.9 | 6 | 0.426 | 0.406 |
| Unigene6933 | PREDICTED: peroxidase 63-like | *Solanum lycopersicum* | 207 | 18 | 5 | 0.577 | 0.484 |
| Unigene9196 | L-ascorbate oxidase | *Nicotiana tabacum* | 532 | 17.9 | 5 | 0.315 | 0.27 |
| **Proteins metabolism (41)** | | | | | | | |
| CL1532.Contig1 | NHP2-like protein 1-like | *Cucumis sativus* | 67 | 7.8 | 1 | 0.416 | 0.363 |
| CL162.Contig1 | 40S ribosomal protein S6, putative | *Ricinus communis* | 198 | 18.8 | 4 | 0.481 | 0.457 |
| CL2014.Contig1 | 40S ribosomal protein S8 | *Zea mays* | 1593 | 47.8 | 6 | 0.656 | 0.657 |
| CL2017.Contig1 | 40S ribosomal protein S11 | *Glycine max* | 79 | 15.1 | 2 | 0.615 | 0.482 |
| CL2035.Contig1 | Perchloric acid soluble translation inhibitor protein | *Arachis hypogaea* | 494 | 42.4 | 6 | 0.651 | 0.618 |
| CL259.Contig2 | 40S ribosomal protein S13 | *Glycine max* | 447 | 39.7 | 4 | 0.653 | 0.507 |
| CL3738.Contig1 | PREDICTED: eukaryotic peptide chain release factor GTP-binding subunit ERF3A-like | *Glycine max* | 157 | 10 | 1 | 0.526 | 0.659 |
| CL3923.Contig2 | PREDICTED: probable peptide transporter At1g52190-like | *Solanum lycopersicum* | 134 | 11.8 | 2 | 0.39 | 0.471 |
| CL3926.Contig2 | 60S ribosomal protein L22-2 | *Arabidopsis thaliana* | 345 | 27.4 | 3 | 0.568 | 0.529 |
| CL4113.Contig2 | tRNA (cytosine-5-)-methyltransferase NSUN2 | *Medicago truncatula* | 419 | 11.1 | 8 | 0.563 | 0.588 |
| CL4489.Contig1 | 39S ribosomal protein L47, mitochondrial-like isoform 1 | *Solanum lycopersicum* | 38 | 12.2 | 2 | 0.437 | 0.392 |
| CL4910.Contig1 | 60S ribosomal protein l10a-1 | *Oryza sativa Indica Group* | 551 | 28.2 | 5 | 0.605 | 0.472 |
| CL516.Contig2 | 60S ribosomal protein L27a-3 | *Arabidopsis thaliana* | 475 | 37.4 | 2 | 0.598 | 0.425 |
| CL5338.Contig1 | 60S ribosomal protein L38 | *Arabidopsis thaliana* | 85 | 18.1 | 2 | 0.584 | 0.446 |
| CL5975.Contig1 | 60S ribosomal protein L35-like | *Fragaria vesca subsp. vesca* | 136 | 31.2 | 1 | 0.524 | 0.394 |
| CL6164.Contig2 | Putative ribosomal RNA methyltransferase NOP2-like | *Vitis vinifera* | 91 | 9.8 | 5 | 0.478 | 0.483 |
| Unigene10001 | PREDICTED: 60S ribosomal protein L5-like | *Cucumis sativus* | 716 | 18.9 | 3 | 0.588 | 0.527 |
| Unigene10012 | 60S ribosomal protein L18a | *Sonneratia alba* | 158 | 15.2 | 4 | 0.459 | 0.285 |
| Unigene1041 | PRA1 family protein B4-like | *Vitis vinifera* | 104 | 11.1 | 2 | 0.571 | 0.5 |
| Unigene11710 | PREDICTED: subtilisin-like protease-like | *Cucumis sativus* | 144 | 11.2 | 5 | 0.203 | 0.195 |
| Unigene12381 | Nascent polypeptide-associated complex subunit alpha-like protein 2 | *Arabidopsis thaliana* | 151 | 21.7 | 3 | 0.61 | 0.442 |
| Unigene12808 | PREDICTED: 60S acidic ribosomal protein P3-like | *Glycine max* | 240 | 22 | 2 | 0.6 | 0.515 |
| Unigene1288 | Ribosomal protein S28e | *Coccomyxa subellipsoidea C-169* | 272 | 18.5 | 1 | 0.542 | 0.605 |
| Unigene12891 | 40S ribosomal protein S21 | *Oryza sativa subsp. japonica* | 583 | 38.6 | 2 | 0.606 | 0.643 |
| Unigene15243 | PREDICTED: nascent polypeptide-associated complex subunit alpha-like | *Vitis vinifera* | 408 | 36.6 | 5 | 0.554 | 0.482 |
| Unigene18463 | 60S ribosomal protein L31-like isoform 1 | *Cucumis sativus* | 155 | 20.8 | 1 | 0.447 | 0.399 |
| Unigene23515 | 60S ribosomal protein L35 | *Euphorbia esula* | 440 | 35.8 | 4 | 0.529 | 0.437 |
| Unigene23525 | 40S ribosomal protein S14 | *Nicotiana tabacum* | 1173 | 57.3 | 7 | 0.624 | 0.58 |
| Unigene242 | DUF246 domain-containing protein At1g04910 | *Arabidopsis thaliana* | 131 | 15.8 | 6 | 0.323 | 0.488 |
| Unigene25697 | 60S ribosomal protein L14-1 | *Arabidopsis thaliana* | 1812 | 34.7 | 4 | 0.553 | 0.65 |
| Unigene26359 | 60S ribosomal protein L6 | *Mesembryanthemum crystallinum* | 797 | 53.8 | 8 | 0.502 | 0.3 |
| Unigene3206 | S-adenosylmethionine-dependent methyltransferase, putative | *Ricinus communis* | 866 | 23.1 | 13 | 0.308 | 0.349 |
| Unigene3929 | 60S acidic ribosomal protein P2, putative | *Ricinus communis* | 310 | 34.8 | 3 | 0.578 | 0.577 |
| Unigene454 | Ribosomal protein P2, putative | *Ricinus communis* | 91 | 10.2 | 4 | 0.489 | 0.595 |
| Unigene3193 | PREDICTED: O-glucosyltransferase rumi homolog | *Vitis vinifera* | 179 | 7.7 | 4 | 0.432 | 0.4 |
| Unigene6596 | 40S ribosomal protein S27-2 | *Arabidopsis thaliana* | 580 | 34.9 | 2 | 0.618 | 0.613 |
| Unigene7293 | 60S ribosomal protein L13 | *Quercus ilex* | 748 | 39.6 | 6 | 0.515 | 0.482 |
| Unigene8897 | Protein disulfide isomerase-like 1-6 | *Arabidopsis thaliana* | 205 | 12.7 | 7 | 0.611 | 0.494 |
| Unigene9125 | PREDICTED: aspartic proteinase nepenthesin-1-like isoform 1 | Vitis vinifera | 339 | 19.1 | 4 | 0.478 | 0.423 |
| Unigene9227 | 60S ribosomal protein L7a | *Oryza sativa subsp. japonica* | 891 | 23 | 7 | 0.611 | 0.391 |
| Unigene9273 | PREDICTED: subtilisin-like protease-like | *Solanum lycopersicum* | 108 | 5 | 2 | 0.369 | 0.44 |
| **Transportation (3)** | | | | | | | |
| CL5170.Contig1 | Sugar transporter type 2a | *Oryza sativa Indica Group* | 92 | 4.7 | 2 | 0.317 | 0.326 |
| CL6046.Contig3 | Calcium-transporting ATPase 8, plasma membrane-type | *Arabidopsis thaliana* | 69 | 1.5 | 2 | 0.421 | 0.377 |
| Unigene3505 | Vesicle-associated protein 3-1 | *Arabidopsis thaliana* | 165 | 18.1 | 3 | 0.536 | 0.594 |
| **Signal transduction (10)** | | | | | | | |
| CL1992.Contig2 | PREDICTED: putative phagocytic receptor 1b-like | *Solanum lycopersicum* | 311 | 14 | 8 | 0.556 | 0.518 |
| CL3257.Contig3 | Brassinosteroid insensitive 1-associated receptor kinase 1 | *Arabidopsis thaliana* | 88 | 12 | 3 | 0.536 | 0.631 |
| CL4493.Contig1 | Probable inactive purple acid phosphatase 29 | *Arabidopsis thaliana* | 64 | 14.8 | 1 | 0.473 | 0.462 |
| CL5097.Contig1 | Auxin-induced in root cultures protein 12 | *Arabidopsis thaliana* | 166 | 13.2 | 2 | 0.498 | 0.608 |
| Unigene1193 | Annexin | *Gossypium hirsutum* | 1298 | 19.9 | 5 | 0.611 | 0.636 |
| Unigene20001 | Putative leucine-rich repeat receptor-like protein kinase | *Arabidopsis thaliana* | 476 | 23.3 | 10 | 0.539 | 0.449 |
| Unigene21232 | PREDICTED: acid phosphatase 1-like | *Solanum lycopersicum* | 142 | 8.3 | 2 | 0.636 | 0.555 |
| Unigene22780 | Probable LRR receptor-like serine/threonine-protein kinase At5g65240 | *Arabidopsis thaliana* | 243 | 14 | 7 | 0.651 | 0.469 |
| Unigene8893 | PREDICTED: polyphosphoinositide phosphatase-like | *Cucumis sativus* | 44 | 3.8 | 3 | 0.639 | 0.614 |
| Unigene9811 | PREDICTED: ADP-ribosylation factor 1-like | *Solanum lycopersicum* | 163 | 37 | 2 | 0.629 | 0.644 |
| **Cell growth/division (22)** | | | | | | | |
| CL3325.Contig2 | Putative DNA ligase | *Pinus sylvestris* | 62 | 6.5 | 4 | 0.594 | 0.481 |
| CL3727.Contig2 | Adenylosuccinate lyase | *Mesembryanthemum crystallinum* | 302 | 18.3 | 1 | 0.484 | 0.649 |
| CL5629.Contig2 | PREDICTED: DNA replication licensing factor MCM4 | *Vitis vinifera* | 161 | 19.3 | 5 | 0.528 | 0.619 |
| CL6066.Contig1 | PREDICTED: condensin complex subunit 3-like | *Fragaria vesca subsp. vesca* | 128 | 2.4 | 2 | 0.582 | 0.511 |
| Unigene10106 | Histone h2a, putative | *Ricinus communis* | 897 | 21.2 | 1 | 0.247 | 0.169 |
| Unigene1032 | Nucleoside diphosphate kinase | *Spinacia oleracea* | 77 | 12.9 | 2 | 0.494 | 0.465 |
| Unigene10378 | DNA replication licensing factor MCM3, putative | *Ricinus communis* | 127 | 9.2 | 1 | 0.468 | 0.464 |
| Unigene10153 | Probable carboxylesterase 7-like | *Fragaria vesca subsp. vesca* | 68 | 26 | 1 | 0.363 | 0.339 |
| Unigene1072 | Licensing factor MCM3, putative | *Ricinus communis* | 88 | 12.5 | 2 | 0.447 | 0.486 |
| Unigene1264 | DNA replication licensing factor MCM7, putative | *Ricinus communis* | 199 | 25.9 | 5 | 0.496 | 0.474 |
| Unigene19874 | Licensing factor MCM7, putative | *Ricinus communis* | 130 | 4.5 | 2 | 0.293 | 0.361 |
| Unigene19924 | PREDICTED: DNA replication licensing factor mcm2 | *Vitis vinifera* | 169 | 22 | 5 | 0.599 | 0.65 |
| Unigene22793 | PREDICTED: DNA replication licensing factor mcm2 | *Vitis vinifera* | 172 | 26.4 | 9 | 0.571 | 0.629 |
| Unigene268 | Dynamin-related protein 1C | *Arabidopsis thaliana* | 793 | 27.9 | 11 | 0.573 | 0.608 |
| Unigene3139 | Histone deacetylase HDT1 | *Arabidopsis thaliana* | 118 | 10.4 | 2 | 0.559 | 0.664 |
| Unigene3571 | PREDICTED: transmembrane 9 superfamily member 4 isoform 1 | *Vitis vinifera* | 845 | 10.9 | 6 | 0.607 | 0.621 |
| Unigene623 | expp1 protein precursor | *Solanum tuberosum* | 143 | 9.7 | 2 | 0.499 | 0.559 |
| Unigene6333 | PREDICTED: DNA replication licensing factor mcm5-A-like | *Vitis vinifera* | 181 | 11.7 | 3 | 0.309 | 0.347 |
| Unigene6719 | Deoxyuridine 5'-triphosphate nucleotidohydrolase | *Arabidopsis thaliana* | 333 | 41.9 | 5 | 0.35 | 0.337 |
| Unigene683 | DNA replication licensing factor MCM3 homolog 2 | *Zea mays* | 202 | 23.8 | 6 | 0.545 | 0.517 |
| Unigene8157 | PREDICTED: DNA replication licensing factor MCM4 | *Vitis vinifera* | 162 | 43 | 3 | 0.336 | 0.554 |
| Unigene9113 | Cell elongation protein diminuto, putative | *Ricinus communis* | 884 | 30.7 | 14 | 0.438 | 0.476 |
| **Cytoskeleton metabolism (22)** | | | | | | | |
| CL1375.Contig2 | Microtubule-associated protein MAP65-1a | *Nicotiana tabacum* | 320 | 13.4 | 6 | 0.442 | 0.495 |
| CL2117.Contig2 | Ankyrin-like protein | *Medicago truncatula* | 713 | 24 | 13 | 0.455 | 0.453 |
| CL2954.Contig2 | Ribosome biogenesis protein WDR12 homolog | *Vitis vinifera* | 36 | 6.4 | 3 | 0.627 | 0.518 |
| CL294.Contig5 | Tubulin beta chain, putative | *Ricinus communis* | 6177 | 53.9 | 8 | 0.533 | 0.592 |
| CL4582.Contig1 | Xyloglucan endotransglucosylase/hydrolase protein 9 | *Arabidopsis thaliana* | 512 | 23.7 | 6 | 0.474 | 0.472 |
| CL54.Contig2 | PREDICTED: cellulose synthase A catalytic subunit 3 [UDP-forming]-like | *Vitis vinifera* | 99 | 3.8 | 3 | 0.541 | 0.666 |
| CL54.Contig4 | Cellulose synthase-3 | *Zea mays* | 167 | 10.8 | 3 | 0.599 | 0.539 |
| CL5933.Contig4 | Xyloglucan endotransglycosylase hydrolase | *Apium graveolens* | 77 | 4.9 | 1 | 0.415 | 0.346 |
| CL880.Contig1 | Probable pectin methyltransferase QUA2 | *Arabidopsis thaliana* | 227 | 17 | 9 | 0.463 | 0.516 |
| Unigene1206 | Mitochondrial import inner membrane translocase subunit | *Solanum nigrum* | 106 | 13.3 | 1 | 0.633 | 0.574 |
| Unigene12854 | PREDICTED: probable histone H2B.1-like | *Fragaria vesca subsp. vesca* | 774 | 38.5 | 3 | 0.571 | 0.517 |
| Unigene15128 | Histone H1 | *Apium graveolens* | 146 | 2.2 | 1 | 0.359 | 0.217 |
| Unigene17241 | Probable galacturonosyl transferase 9 | *Arabidopsis thaliana* | 117 | 7.9 | 4 | 0.513 | 0.527 |
| Unigene17275 | Monocopper oxidase-like protein SKU5-like | *Fragaria vesca subsp. vesca* | 785 | 19.1 | 8 | 0.56 | 0.637 |
| Unigene17794 | Pistil-specific extensin-like protein | *Nicotiana tabacum* | 85 | 11.5 | 2 | 0.396 | 0.423 |
| Unigene19881 | PREDICTED: reticulon-like protein B1-like | *Solanum lycopersicum* | 189 | 14.6 | 3 | 0.492 | 0.516 |
| Unigene25391 | Glucomannan 4-beta-mannosyltransferase | *Arabidopsis thaliana* | 50 | 8.4 | 4 | 0.35 | 0.458 |
| Unigene29885 | Pectin acetylesterase | *Eucalyptus globulus subsp. globulus* | 238 | 36 | 6 | 0.543 | 0.404 |
| Unigene4028 | PREDICTED: expansin-like A1-like | *Fragaria vesca subsp. vesca* | 200 | 17.8 | 4 | 0.499 | 0.378 |
| Unigene477 | Histone H1E | *Nicotiana tabacum* | 48 | 15.8 | 2 | 0.42 | 0.444 |
| Unigene561 | PREDICTED: endoglucanase 12-like, partial | *Vitis vinifera* | 198 | 9.2 | 4 | 0.46 | 0.416 |
| Unigene6331 | Putative xyloglucan endotransglycosylase, partial | *Bassia scoparia* | 172 | 14.3 | 4 | 0.539 | 0.488 |
| **Metabolism (11)** | | | | | | | |
| CL3148.Contig2 | Probable methyltransferase PMT26 | *Arabidopsis thaliana* | 708 | 26.8 | 16 | 0.435 | 0.446 |
| CL3803.Contig2 | phosphoethanolamine N-methyltransferase | *Suaeda japonica* | 496 | 18.9 | 8 | 0.364 | 0.484 |
| CL4460.Contig2 | Phospholipase C 3 | *Mycobacterium tuberculosis* | 192 | 11.8 | 4 | 0.581 | 0.657 |
| CL4465.Contig2 | NADH: nitrate reductase, partial | *Spinacia oleracea* | 325 | 15.8 | 12 | 0.468 | 0.546 |
| Unigene11316 | PREDICTED: probable methyltransferase PMT2-like | *Fragaria vesca subsp. vesca* | 992 | 37.2 | 18 | 0.47 | 0.481 |
| Unigene11757 | Probable methyltransferase PMT21 | *Arabidopsis thaliana* | 1071 | 36.2 | 13 | 0.398 | 0.381 |
| Unigene19705 | Synaptic glycoprotein sc2 | *Oryza sativa Indica Group* | 139 | 12.6 | 3 | 0.598 | 0.457 |
| Unigene225 | Probable methyltransferase PMT9 | *Arabidopsis thaliana* | 111 | 10.2 | 4 | 0.6 | 0.612 |
| Unigene22884 | Short-chain dehydrogenase/reductase | *Populus tremula* | 169 | 20 | 6 | 0.581 | 0.602 |
| Unigene25919 | NADP-specific isocitrate dehydrogenase | *Eriobotrya japonica* | 48 | 4.5 | 2 | 0.434 | 0.438 |
| Unigene9112 | PREDICTED: probable methyltransferase PMT13-like | *Solanum lycopersicum* | 173 | 8.9 | 4 | 0.434 | 0.434 |
| **Secondary metabolism (5)** | | | | | | | |
| CL957.Contig1 | PREDICTED: brassinosteroid-regulated protein BRU1 | *Vitis vinifera* | 118 | 13.3 | 2 | 0.437 | 0.526 |
| Unigene17954 | Obtusifoliol-14-demethylase | *Gossypium hirsutum* | 366 | 19.5 | 6 | 0.492 | 0.531 |
| Unigene1977 | CXE carboxylesterase | *Malus pumila* | 119 | 20 | 2 | 0.379 | 0.389 |
| Unigene23131 | PREDICTED: 3-ketoacyl-CoA synthase 11-like isoform 2 | *Glycine max* | 159 | 9.8 | 4 | 0.528 | 0.626 |
| Unigene25703 | PREDICTED: cytochrome P450 86A1-like | *Cucumis sativus* | 37 | 2.1 | 1 | 0.382 | 0.345 |
| **Transcription (16)** | | | | | | | |
| CL2095.Contig2 | PREDICTED: DNA-directed RNA polymerase I subunit RPA2 | *Vitis vinifera* | 110 | 4.6 | 4 | 0.505 | 0.603 |
| CL4280.Contig2 | PREDICTED: pre-mRNA-splicing factor SF2-like | *Solanum lycopersicum* | 54 | 10.7 | 3 | 0.617 | 0.632 |
| CL4683.Contig2 | PREDICTED: U1 small nuclear ribonucleoprotein 70 kDa-like | *Vitis vinifera* | 74 | 5.4 | 2 | 0.588 | 0.58 |
| CL4898.Contig1 | Probable small nuclear ribonucleoprotein | *Arabidopsis thaliana* | 203 | 27.3 | 2 | 0.634 | 0.585 |
| CL916.Contig2 | PREDICTED: small nuclear ribonucleoprotein Sm D3-like | *Fragaria vesca subsp. vesca* | 96 | 9.7 | 1 | 0.406 | 0.274 |
| Unigene12044 | DEAD-box ATP-dependent RNA helicase 7 | *Spinacia oleracea* | 140 | 14.5 | 8 | 0.564 | 0.512 |
| Unigene15074 | Chromatin binding protein, putative | *Ricinus communis* | 40 | 2.8 | 1 | 0.661 | 0.539 |
| Unigene15685 | PREDICTED: U1 small nuclear ribonucleoprotein A | *Vitis vinifera* | 131 | 20.6 | 4 | 0.452 | 0.552 |
| Unigene17446 | Small nuclear ribonucleoprotein polypeptide | *Jatropha curcas* | 129 | 36.4 | 3 | 0.541 | 0.442 |
| Unigene20635 | PREDICTED: heterogeneous nuclear ribonucleoprotein F-like | *Solanum lycopersicum* | 104 | 3.2 | 1 | 0.617 | 0.557 |
| Unigene25404 | PREDICTED: putative chromatin-remodeling complex ATPase chain-like isoform 2 | *Glycine max* | 188 | 7.2 | 6 | 0.587 | 0.615 |
| Unigene25902 | DEAD-box ATP-dependent RNA helicase 28 | *Oryza sativa subsp. japonica* | 111 | 4.3 | 3 | 0.382 | 0.328 |
| Unigene26787 | PREDICTED: DNA-directed RNA polymerases I, II, and III subunit | *Vitis vinifera* | 59 | 28.2 | 2 | 0.607 | 0.631 |
| Unigene3779 | PREDICTED: elongator complex protein 3-like | *Solanum lycopersicum* | 146 | 11.6 | 5 | 0.541 | 0.574 |
| Unigene8836 | Nuclear transcription factor Y subunit C-9 | *Medicago truncatula* | 85 | 5.2 | 1 | 0.57 | 0.505 |
| Unigene9666 | PREDICTED: protein mago nashi homolog isoform 2 | *Cucumis sativus* | 64 | 33.6 | 3 | 0.621 | 0.599 |
| **Unclassified (6)** | | | | | | | |
| CL1706.Contig2 | PREDICTED: HIPL1 protein-like | *Vitis vinifera* | 180 | 16.6 | 9 | 0.483 | 0.467 |
| Unigene11846 | Pentatricopeptide repeat-containing protein At4g36680, mitochondrial | *Arabidopsis thaliana* | 98 | 13.1 | 4 | 0.401 | 0.455 |
| Unigene14547 | PREDICTED: protein CfxQ-like isoform 1 | *Solanum lycopersicum* | 318 | 14.2 | 5 | 0.584 | 0.592 |
| Unigene17655 | TPR domain containing protein | *Zea mays* | 357 | 16.7 | 6 | 0.584 | 0.58 |
| Unigene6561 | Chemocyanin | *Lilium longiflorum* | 198 | 9.3 | 1 | 0.223 | 0.196 |
| Unigene6823 | putative CPF 0172 family protein | *Salicornia bigelovii* | 473 | 44.9 | 7 | 0.596 | 0.585 |
| **Unknown (24)** | | | | | | | |
| CL1303.Contig2 | Hypothetical protein VITISV_026367 | *Vitis vinifera* | 319 | 24.4 | 4 | 0.59 | 0.604 |
| CL1481.Contig1 | PREDICTED: uncharacterized protein LOC100267719 isoform 1 | *Vitis vinifera* | 99 | 10.3 | 2 | 0.397 | 0.418 |
| CL2596.Contig3 | Hypothetical protein VITISV_033728 | *Vitis vinifera* | 237 | 2.2 | 1 | 0.46 | 0.537 |
| CL2652.Contig1 | Conserved hypothetical protein | *Ricinus communis* | 92 | 4 | 1 | 0.596 | 0.618 |
| CL2732.Contig2 | PREDICTED: protein RRP5 homolog | *Vitis vinifera* | 159 | 3.7 | 4 | 0.527 | 0.467 |
| CL382.Contig5 | Unnamed protein product | *Vitis vinifera* | 262 | 16.7 | 5 | 0.606 | 0.547 |
| CL6073.Contig1 | Unnamed protein product | *Vitis vinifera* | 123 | 9.6 | 4 | 0.412 | 0.421 |
| Unigene12373 | Predicted protein | *Populus trichocarpa* | 92 | 18.3 | 3 | 0.382 | 0.573 |
| Unigene12678 | Hypothetical protein OsJ_27896 | *Oryza sativa Japonica Group* | 105 | 15.2 | 3 | 0.463 | 0.574 |
| Unigene14637 | Unnamed protein product | *Vitis vinifera* | 128 | 21.9 | 5 | 0.473 | 0.602 |
| Unigene18431 | PREDICTED: uncharacterized protein LOC100262248 | *Vitis vinifera* | 118 | 20.5 | 1 | 0.648 | 0.585 |
| Unigene20324 | Predicted protein | Populus trichocarpa | 297 | 10.4 | 4 | 0.505 | 0.494 |
| Unigene20771 | PREDICTED: uncharacterized protein LOC101307237 | *Fragaria vesca subsp. vesca* | 79 | 8.5 | 1 | 0.365 | 0.472 |
| Unigene22849 | Unnamed protein product | *Vitis vinifera* | 156 | 17.3 | 6 | 0.543 | 0.587 |
| Unigene22994 | Hypothetical protein RCOM_1499820 | *Ricinus communis* | 242 | 12.3 | 3 | 0.198 | 0.201 |
| Unigene23166 | Predicted protein | *Populus trichocarpa* | 280 | 20.2 | 6 | 0.519 | 0.634 |
| Unigene23316 | Uncharacterized protein LOC100250699 | *Vitis vinifera* | 536 | 26.5 | 9 | 0.482 | 0.498 |
| Unigene23460 | Unknown | *Medicago truncatula* | 192 | 11 | 4 | 0.588 | 0.508 |
| Unigene25160 | Hypothetical protein PRUPE_ppa006690mg | *Prunus persica* | 140 | 11 | 4 | 0.478 | 0.649 |
| Unigene339 | Unnamed protein product | *Vitis vinifera* | 227 | 18.5 | 4 | 0.568 | 0.445 |
| Unigene3982 | Hypothetical protein VITISV_039073 | *Vitis vinifera* | 519 | 16.8 | 5 | 0.608 | 0.593 |
| Unigene572 | Hypothetical protein PRUPE_ppa008602mg | *Prunus persica* | 84 | 8.9 | 3 | 0.542 | 0.534 |
| Unigene6611 | Predicted protein | *Populus trichocarpa* | 93 | 11.5 | 2 | 0.563 | 0.598 |
| Unigene6620 | Predicted protein | *Populus trichocarpa* | 224 | 15.9 | 5 | 0.647 | 0.656 |

^a^Accession no. according to EST database of Halogeton glomeratus.

^b^The values were calculated as the ratio between intensities of identified protein in treatments (200, and 400 mM) vs ck (0 mM NaCl)
