# Supplementary material for: Comparative Proteomic Analysis of Cultured Suspension Cells of the Halophyte Halogeton glomeratus by iTRAQ Provides Insights into Response Mechanisms to Salt Stress
Source: Front Plant Sci. 2016 Feb 9;7:110. doi: 10.3389/fpls.2016.00110 (PMC4746295; doi:10.3389/fpls.2016.00110)
Supplement: Supplementary file 3 [file Table3.doc]

Table S2. Proteins identified as involved in regulating Na^+^ and K^+^ homeostasis under salt stress.

| Accession  no^a^. | Protein name | Species | Score | Coverage (%) | Peptides | Ratio^b^ | |
| --- | --- | --- | --- | --- | --- | --- | --- |
|  |  |  |  |  |  | 200 | 400 |
| **Vacuolar ATPase (9)** | | | | | | | |
| CL2680.Contig2 | Vacuolar H^+^-ATPase subunit A | *Mesembryanthemum crystallinum* | 9625 | 69.7 | 39 | 1.144 | 1.066 |
| Unigene20250 | V-type proton ATPase subunit E | *Spinacia oleracea* | 883 | 34.3 | 9 | 1.116 | 0.875 |
| CL4328.Contig2 | Vacuolar-type H^+^-ATPase subunit B2 | *Arabidopsis thaliana* | 5909 | 54.6 | 21 | 1.162 | 1.111 |
| CL5130.Contig1 | Vacuolar proton pump ATPase subunit H | *Suaeda corniculata* | 1876 | 39.8 | 15 | 1.011 | 0.95 |
| CL2727.Contig1 | Vacuolar proton-pumping PPase | Oxybasis rubra | 516 | 8.4 | 5 | 0.962 | 1.029 |
| Unigene25805 | V-type proton ATPase subunit d2 | *Arabidopsis thaliana* | 505 | 20.5 | 7 | 1.008 | 1.023 |
| Unigene6572 | V-type proton ATPase subunit F | *Arabidopsis thaliana* | 102 | 56.6 | 2 | 1.577 | 1.277 |
| Unigene17604 | Vacuolar proton-ATPase subunit -like | *Arabidopsis thaliana* | 44 | 2.5 | 1 |  |  |
| Unigene15953 | V-type H^+^-transporting ATPase subunit b | *Galdieria sulphuraria* | 34 | 5.7 | 1 |  |  |
| **Plasma membrane ATPase (3)** | | | | | | | |
| CL510.Contig4 | plasma membrane H^+^-ATPase | *Sesuvium portulacastrum* | 2796 | 35.5 | 13 | 0.789 | 0.71 |
| CL510.Contig3 | PREDICTED: ATPase 11, plasma membrane-type | *Vitis vinifera* | 1430 | 28.5 | 13 | 0.709 | 0.854 |
| Unigene3448 | plasma membrane H^+^-ATPase | *Sesuvium portulacastrum* | 198 | 15.6 | 3 | 0.737 | 0.785 |
| **Antiporter (6)** | | | | | | | |
| CL4432.Contig2 | potassium efflux antiporter | *Populus trichocarpa* | 596 | 9.7 | 8 | 1.108 | 1.169 |
| Unigene23705 | putative potassium transporter KUP3, partial | *Alternanthera philoxeroides* | 192 | 14.3 | 3 | 0.883 | 0.825 |
| CL3628.Contig2 | salt overly sensitive 1B | *Chenopodium quinoa* | 75 | 5.1 | 2 | 0.765 | 0.711 |
| Unigene1350 | PREDICTED: cation-chloride cotransporter 1 isoform 2 | *Vitis vinifera* | 37 | 8.2 | 1 |  |  |
| CL3987.Contig1 | putative potassium transporter KUP3, partial | *Alternanthera philoxeroides* | 36 | 8.8 | 1 |  |  |
| Unigene3263 | Na^+^/H^+^ antiporter | *Mesembryanthemum crystallinum* | 16 | 1.3 | 1 |  |  |
| **Ion channel (3)** | | | | | | | |
| Unigene14876 | voltage-dependent anion channel | *Beta vulgaris* | 2393 | 33.7 | 8 | 1.128 | 0.955 |
| Unigene704 | voltage-dependent anion-selective channel, putative | *Ricinus communis* | 1210 | 54.0 | 10 | 1.091 | 0.897 |
| CL2857.Contig1_All | potassium channel beta subunit | *Platanus x acerifolia* | 611 | 37.2 | 10 | 0.845 | 0.878 |

^a^Accession no. according to EST database of *Halogeton glomeratus*.

^b^The values were calculated as the ratio between intensities of identified protein in treatments (200, and 400 mM) vs ck (0 mM NaCl)
